# A Rational Framework for Group-Based Selective Social Learning

**DOI:** 10.1162/opmi_a_00205

**Published:** 2025-05-09

**Authors:** Max Taylor-Davies, Neil Bramley, Christopher G. Lucas

**Affiliations:** School of Informatics, University of Edinburgh, Edinburgh, UK

**Keywords:** social learning, social learning strategies, preference inference, theory of mind, rational analysis, Bayesian models

## Abstract

Social learning can be a powerful tool, allowing us to acquire knowledge and adaptive behaviours while bypassing many of the costs of learning through direct experience. However, not everyone’s behaviour is equally valuable to learn from, as other people’s goals or preferences may differ dramatically from our own. In this paper, we consider the problem of selectively learning from others on the basis of direct and indirect inferences about their task-relevant preferences. Specifically, we focus on the setting where a social learner must generalise preference judgements across individuals using shared features and other cues, and so develop a formal account that can reconcile a seemingly disparate empirical picture of group-based selective social learning. Across three behavioural experiments, we demonstrate that people are sensitive to the contextual significance of group identity cues when choosing who to learn from in partially observed environments. We show that this behaviour cannot be accounted for by a range of simpler heuristic strategies.

## INTRODUCTION

In navigating an open-ended and changing environment, we frequently encounter new problems where we must make decisions on the basis of limited information. One tool we rely on to make better decisions is *social learning*; that is, adopting adaptive behaviours based on information from others in addition to or as a substitute for first-hand experience (Gelpí & Buchsbaum, 2023; Henrich, 2018). But we do not imitate or learn from others indiscriminately; we are *selective* in choosing whom to learn from (Boyd & Richerson, 1985; Laland, 2004; Mesoudi & O’Brien, 2008; Rendell et al., 2010; Rogers, 1988).

Most of these selection strategies focus on attributes of individual demonstrators (such as their competence or social status). The role of group identity in selective social learning is less well-understood. Prior work points to the existence of an ingroup bias, where people learn preferentially from members of their own social group (Buttelmann et al., 2013; Golkar et al., 2015; Howard et al., 2015; Pető et al., 2018). This bias has been found even in a minimal-groups setting, suggesting that the effect is not merely a result of other factors such as familiarity (Montrey & Shultz, 2022). At the same time, other research has demonstrated cases where people learn preferentially from members of the outgroup, such as children favouring information from teachers rather than from their peers (Rakoczy et al., 2010; Seehagen & Herbert, 2011). This indicates a role for group identity that is not only limited to supporting ingroup bias, with learners being sensitive (at least in some cases) to the relative suitability of different groups as sources of information or models of behaviour.

In this paper, we use the little-explored angle of learner and demonstrator *preferences* to study whether people can make flexible use of group identity cues in selecting social learning targets. One potential rationalisation given for ingroup-biased social learning is that individuals from the same social group are often more likely to have shared preferences. But a *rational* social learner should also recognise when this is not the case, and choose demonstrators based on evidence of how different groups’ preferences relate to their own—rather than blindly adhering to a ‘copy-the-ingroup’ strategy. Through a series of experiments, we develop and illustrate a rational model of group-based selective social learning that successfully captures people’s behaviour in a virtual decision-making environment.

The paper is organised as follows. First, we outline the general case for social learning as a complement to individual learning, and for selective over indiscriminate social learning. We then formalise the specific problem of social learning under *preference variation*, and outline what a rational (utility-maximising) strategy for selecting social learning targets should look like. Focusing first on the case where the learner has *direct* evidence of other agents’ choice behaviour, we draw a connection with existing literature on the rational inference of preferences from behaviour. In Experiment 1, we show that people can use rational preference inference to select social learning targets. We next consider the general problem of learning from *unfamiliar* agents, whose behaviour we have never directly observed, using non-behavioural features to generalize their likely preferences from past encounters with other agents. We discuss the idea that people can use inferences to the presence of latent social groups with similar preferences to select suitable learning targets based on their individual behavioural or featural cues. We formalise this idea within a hierarchical Bayesian model, and validate its predictions with a second experiment. Concretely, we show this model can predict the social learning choices of participants across a series of decision-making tasks with partial environmental and social information. Finally, we report the results of a third Experiment which adds an element of context-dependence, where rational social learners should learn from agents in different groups depending on the features of the choice they face. We conclude by discussing the implications of our results, and highlighting promising directions for future research.

## DECISION-MAKING, UNCERTAINTY AND SOCIAL LEARNING

As we go about our lives, we frequently have to make choices between different options or actions. Some of these choices are inconsequential: what to eat for lunch, which brand of shampoo to buy. Others are more significant, such as which city to live in or what career to pursue. Sometimes, we are lucky enough to have all the information we need to make a choice—but more often we must decide under some degree of ignorance or uncertainty. This can take different forms, but generally boils down to incomplete knowledge about our environment: we may not be aware of what options are available to us; or if we are, we may not be able to predict the consequences of a given choice. Faced with this prospect, a naïve strategy is simply to try every available path or possibility. Unfortunately, this approach is not always viable. The space of possible actions and their potential consequences is often so large as to make it impossible to thoroughly test the waters, especially if operating under limited time or cognitive capacity. What’s more, even if the space of options is small, some might be risky—for our ancestors, a taste of the wrong mushroom or berry could end very badly.

An alternative approach is to learn *socially*. Being guided by the choices of agents who are more knowledgeable or better adapted to the environment allows us to avoid many of the costs and risks involved in individual trial-and-error learning. While most adults know not to walk into traffic or drink cleaning products, an infant’s environment is marked by uncertainty and risk, making social learning especially critical in early development. But even into adulthood, we often seek the guidance of others in unfamiliar domains—such as in our mushroom foraging example, where social and cultural learning are found to play a central role (Kaaronen, 2020; Kaaronen et al., 2023). The argument for social learning as a shortcut through difficult search or exploration problems accords well with ‘resource-rational’ models of cognition, which have gained traction in recent years and hold that people pursue strategies which are optimal with respect to tradeoffs between their expected rewards and the cognitive effort or computational costs involved (Bhui et al., 2021; Icard, 2023; Lai & Gershman, 2024; Lieder & Griffiths, 2019). Under such a framing, social learning could be viewed as an attractive option when exhaustive exploration or deliberation is possible but computationally expensive.

### Idealised Social Learning

Before going further, it is helpful to briefly formalise the general problem of learning adaptive behaviour. To do this, we use the framework of Markov Decision Processes (MDPs; for a comprehensive overview, see Puterman, 1994; Sutton & Barto, 2018), a common choice for studying sequential decision-making problems. An MDP is described by a state space 𝒮, an action space 𝒜, a (stochastic) transition function *T* : 𝒮 × 𝒜 × 𝒮 → [0, 1], and a reward function *R* : 𝒮 × 𝒜 → ℝ. An agent within an MDP acts according to a stochastic *policy* *π* : 𝒮 × 𝒜 → [0, 1], where *π*(*s*, *a*) gives the probability of taking action *a* in state *s* (these definitions are also given in Appendix A). For a given policy *π*, the action-value function (*Q*) is given by

$$Q_{\pi }\left(s,a\right)={𝔼}_{\pi }\left[G_{t}|S_{t}=s,A_{t}=a\right]$$ (1)

where *G*_*t*_ = *R*_*t*+1_ + *γR*_*t*+2_ + *γ*^2^*R*_*t*+3_ + … is the discounted expected future return from timestep *t*, i.e., the sum of all future rewards discounted by *γ* ∈ [0, 1). An MDP is ‘solved’ by finding optimal policy π* that maximises this return.

One large class of methods for solving MDPs is given by the field of reinforcement learning (RL) (Arulkumaran et al., 2017; François-Lavet et al., 2018; Kober et al., 2013; Sutton & Barto, 2018), which involves estimating π* via repeated trial-and-error interaction between agent and environment. While many RL algorithms have been proposed, a dominant class are ‘value-based’, meaning they approximate the action-value function $\hat{Q}$ and use it to guide a greedy policy such as *π*(*s*, *a*) ∝ 𝟙[*a* = argmax_*a*^′^_ $\hat{Q}$(*s*, *a*′)], where 𝟙 is the indicator function. In these methods, $\hat{Q}$ is learned through direct interaction with the environment. But as we highlight above, this is not always safe or feasible—in some environments, taking the wrong action can have permanent consequences; in others, the sheer number of possible choices can overwhelm a naïve explorer. Instead, we would in some cases like to learn π* *socially*, given a state-action observation history **O** = {(*s*, *a*)} of other agents’ behaviour. Of course, what we can learn from **O** depends on what we know about the policies of the agents we have observed. In general, the space of possible policies a given agent might follow is so large that understanding their actions would be effectively impossible without suitable inductive biases to constrain explanations. One simplifying assumption that human learners seem to rely on is to treat other agents as *approximately rational* (Baker et al., 2017; Dennett, 1987). Specifically, we assume a rational social learner models other agents as selecting actions according to a noisily reward-maximising “Boltzmann” (aka softmax) policy given by

$$\pi_{\text{bol}}\left(s,a;Q,\beta \right)\propto \exp\left(\frac{Q\left(s,a\right)}{\beta }\right)$$ (2)

While this is far from the only way to model boundedly rational agents, and indeed recent work has highlighted certain shortcomings (Alanqary et al., 2021; Bobu et al., 2020; Evans et al., 2016; Zhi-Xuan et al., 2020), its simplicity and tractability as an approach has rendered it attractive for a wide range of work in both cognitive modelling (Baker et al., 2009; Goodman et al., 2009; Jara-Ettinger et al., 2016) and AI (Finn et al., 2016; Ramachandran & Amir, 2007; Ziebart et al., 2008). In addition, we will assume that all agents other than the social learner are following a stationary policy, i.e., they are not currently learning themselves.

To infer the action-value function $\hat{Q}$ from observation, and thereby learn what actions will be more or less rewarding in each state, we could simply invert Equation 2 (assuming at least a rough idea of our target agent’s *β* and *γ* parameters)—essentially asking ‘what goals or preferences would have led a rational agent to produce this behaviour?’:

$${\hat{Q}}^{*}=\underset{Q^{\prime }\in Q}{\mathrm{argmax}}\prod_{\left(s,a\right)\in O}\pi_{\text{bol}}\left(s,a;Q^{\prime },\beta \right)$$ (3)

This kind of inverse social reasoning approach, which draws on the longstanding idea that people perceive the actions of others as fundamentally *intentional* (Dennett, 1987; Gergely et al., 1995; Gopnik & Meltzoff, 1997; Heider & Simmel, 1944), has been formalised in recent years through models of Bayesian inference (Baker et al., 2009, 2017; Jern et al., 2017; Lucas et al., 2009, 2014; Ullman et al., 2009). It also bears a strong relation to the paradigm of *inverse reinforcement learning* (IRL) (Jara-Ettinger, 2019; Ramachandran & Amir, 2007; Ziebart et al., 2008). By reverse-engineering a policy in this manner, an agent can learn the value of different actions without having to suffer the ill effects of making bad choices in a dangerous environment.

### Bounded Social Learning

But while the above approach would seem to address the risk minimisation argument for social learning, recall that our other motivation was grounded in cognitive cost. Looking at Equation 3, we face an optimisation problem over the set of possible *Q*-functions. Solving this in practice typically involves repeated solving of the forward RL problem (Ho & Ermon, 2016; Ramachandran & Amir, 2007; Ziebart et al., 2008), which can become very computationally expensive. To avoid this, we could instead follow a heuristic approach that just *copies behaviour directly* from the observed agents by sampling from **O**. This is certainly a cheaper approach, at least where **O** is small enough for the agent to store in memory, replacing the optimisation problem with straightforward sampling—but it does have drawbacks that are worth highlighting. First, the success of this heuristic is bounded by the competence of the agent(s) whose behaviour we copy; second, we miss out on the opportunity to use *Q* to generalise to previously unseen state-action pairs.

These drawbacks mean that simple imitation may in general be less effective as a social learning method than fully fledged IRL. However, we will consider here only settings where direct imitation has no disadvantage relative to an IRL approach (i.e., all observed agents are perfectly rational, and the learner’s observation history always gives adequate coverage of the state space). Our rationale for doing this is as follows. We might think of an end-to-end selective social learning policy as consisting of two stages, where the first stage maps from observations of agents and environment to a choice of demonstrator, and the second maps from this choice to actual actions for the learner to execute. In the current work, we are concerned only with the first stage; i.e., with the problem of *whom* to learn from, not *how* to learn from them. Since imitation is conceptually simpler and easier to model, that’s the approach we adopt; but we make no particular claim about whether it’s in general the best choice for modelling this second stage. Future work might consider a more complete model of social learning that accounts for non-imitative approaches such as emulation or socially-guided exploration (Vélez et al., 2022; Witt et al., 2024).

## CHOOSING *WHOM* TO LEARN FROM

While social learning can be a powerful vehicle for agents to acquire adaptive behaviour while avoiding some of the costs of learning asocially, the use of social information is not in itself a guarantee of success. The general principle that agents should engage in *selective* rather than *indiscriminate* social learning has long been recognised within the literature of fields such as psychology, behavioural ecology and evolutionary biology (Boyd & Richerson, 1985; Mesoudi & O’Brien, 2008; Rendell et al., 2010; Rogers, 1988). Our focus here is on work that considers the specific question of *whom* to learn from. An early presentation of the idea that a demonstrator’s identity can impact their salience to an observer is given by Coussi-Korbel and Fragaszy (1995) under the name of ‘directed social learning‘. The authors suggest that directed social learning enables individuals to adapt more flexibly to a changing environment, by improving the efficiency of information transmission within a social group. Across both humans and nonhuman animals, evidence has been found for a number of strategies for directed social learning. The majority of these are simple heuristics for guiding social learners to more successful individuals—either through direct evidence of success or competence (Birch et al., 2008; Brody & Stoneman, 1985; Jaswal & Malone, 2007; Wilkinson, 1992; Zmyj et al., 2010), or using indirect cues such as age or social rank (Abramovitch et al., 1980; Duffy et al., 2009; Horner et al., 2010; Jaswal & Neely, 2006; Rakoczy et al., 2010; Seehagen & Herbert, 2011). Social learners may also be guided by the amount of attention paid to a particular demonstrator by others—typically referred to as prestige bias (Chudek et al., 2012; Henrich & Gil-White, 2001).

However, strategies aimed towards learning from more proficient or successful demonstrators, while clearly powerful, are insufficient to capture the full space of adaptive social learning behaviour. By focusing solely on competence, they ignore another key way in which agents can differ: their *values* or *preferences*. Of course, this focus makes sense within many of the contexts typically considered in work on social learning strategies. In a foraging context, any given set of stickleback fish will typically share the basic value that locations yielding more food are to be preferred—and so a demonstrator’s foraging skill is the only relevant factor (Duffy et al., 2009). Or in our own earlier example, the mushrooms that are poisonous to a child learner will also be poisonous to anyone whose choices they might copy; and so selecting the most knowledgeable social learning target is sufficient to achieve optimal outcomes. But in many scenarios that involve human decision-making under uncertainty, these assumptions don’t necessarily hold: a vegan ordering in a restaurant will generally not want to copy the choices of a committed carnivore just because they have better knowledge of the menu, and a person with deeply held socialist beliefs would not want to follow the voting behaviour of someone on the far right just because they have more information about the candidates. Any complete theory of selective social learning in humans will thus need to offer an account of how people navigate these differences in values or preferences. Prior work offers some evidence for preferences playing a role in human social learning, with Fawcett and Markson (2010) finding that children as young as 2 years old can reason specifically about the extent to which another agent *shares* their preferences, and use this to guide their own choice behaviour under limited information—but a broader formal account is still lacking.

In the subsequent sections of the current work, we develop such an account at the level of both individual agents and social groups, and provide empirical evidence for preference-guided selectivity in adult social learning. First, in the tradition of rational analysis (Anderson, 1990), we consider what an optimal approach to preference-based selective social learning might look like. We then report the results of a first Experiment showing that this normative strategy successfully captures people’s behaviour in a virtual decision-making task. Following this, we develop our account from the level of individuals to a setting that focuses on social group structure rather than dyadic similarity.

## OPTIMAL SELECTIVE SOCIAL LEARNING

To formalise the problem of selective social learning under preference variation, we make a slight extension to the MDP setup outlined in Decision-Making, Uncertainty and Social Learning section: rather than defining a global reward function *R*, we instead say that every agent *m* has their own internal *utility function* *U*^(*m*)^ : 𝒮 × 𝒜 → ℝ. Depending on the setting, these utility functions might capture direct external rewards/penalties imposed by the environment, agents’ intrinsic motivations, or some combination of both. The reward that an agent with utility function *U*^(*m*)^ receives for executing a trajectory *τ* (i.e., a sequence of actions) of length *T* is then given by *R*(*τ*; *U*^(*m*)^) = $\sum_{t=1}^{T}$ *γ*^*t*−1^*U*^(*m*)^(*S*_*t*_, *A*_*t*_) where *S*_*t*_, *A*_*t*_ are the state and action at time *t*. The relaxation from a global reward function to a set of agent-specific utility functions also means that expected state-action values now vary between agents, i.e., *Q*^(*m*)^(*s*, *a*) = *Q*(*s*, *a*∣*U*^(*m*)^). Aside from this, we assume agents follow the same form of approximately rational^1^ policy as before, i.e.,

$$\pi^{\left(m\right)}\left(s,a\right)\propto \exp\left(\frac{Q^{\left(m\right)}\left(s,a\right)}{\beta^{\left(m\right)}}\right)$$ (4)

**Figure 1.**
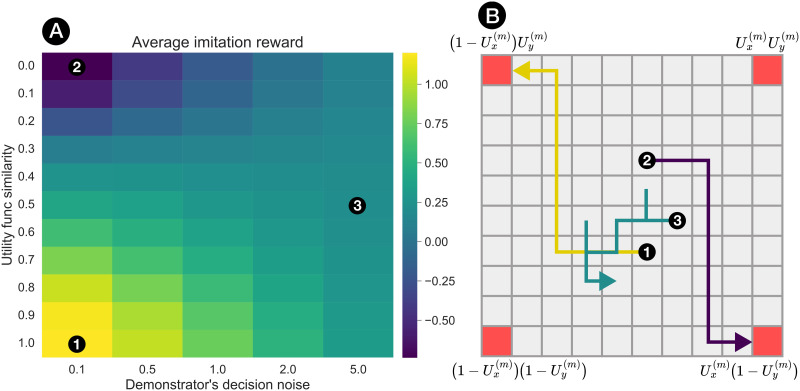
(A) Average return from imitating in a simple 4-goal gridworld environment as a function of the similarity between the imitator’s and demonstrator’s utility functions, and the demonstrator’s decision noise. At each simulation trial, each demonstrator agent executes a noisy trajectory from a randomised starting location to one of the corner tiles (highlighted in red), following a Boltzmann policy (Equation 4) governed by their 2D utility function and level of decision noise (and with a small fixed cost per step). We then compute the average return over each demonstrator’s trajectory history under the *imitator’s* utility function. Shown in (B) are example trajectories for demonstrators at 3 points on the return surface, for an imitator’s utility function that assigns positive value only to the top-left corner (i.e., *u*_*x*_ < 0, *u*_*y*_ > 0) and with the high-noise turquoise trajectory truncated at 10 steps.

We now introduce to the environment a social learner, the ‘ego agent’ (equivalent to the participant in the behavioural experiments we describe later), who wants to execute actions that maximise their own utility function *U*^ego^ while avoiding trial-and-error exploration. Instead, they will simply copy one of the other agents present—that is, they will select an agent *m*, reproduce exactly the trajectory *τ*^(*m*)^ that agent *m* then executes, and consequently receive some reward determined by *U*^ego^. To maximise their expected return, the social learner should select an agent m* = argmax_*m*_ 𝔼[*R*(*τ*^(*m*)^; *U*^ego^)], i.e., copy the agent whose behaviour will be most rewarding (in expectation) under *U*^ego^. Intuitively, the extent to which agent A’s trajectory is rewarding for agent B should depend on two factors: how competent agent A is, and how *similar* the two agents’ utility functions are. That is,

$$𝔼\left[R\left(\tau^{\left(m\right)};U^{\text{ego}}\right)\right]\propto \text{sim}\left(U^{\text{ego}},U^{\left(m\right)}\right)𝔼\left[R\left(\tau^{\left(m\right)};U^{\left(m\right)}\right)\right]$$ (5)

where sim is some function that captures the similarity between two utility functions over dimensions relevant to the current decision-making context^2^. This intuition is illustrated in Figure 1, using a simple four-goal gridworld environment (which we will go on to extend for our experiments), and a similarity function given by sim(*U*^ego^, *U*^(*m*)^) = ∑_*i*_ ∣$U_{i}^{\text{ego}}$ − 0.5∣(1 − ∣$U_{i}^{\text{ego}}$ − $U_{i}^{\left(m\right)}$∣) with *U*_*i*_ ∈ [0, 1].

If we now assume that all potential demonstrators are equally competent (e.g., the leftmost column of the heatmap in Figure 1A), optimal selection simplifies to

$$m^{*}=\underset{m}{\mathrm{argmax}}\;\text{sim}\left(U^{\text{ego}},U^{\left(m\right)}\right)$$ (6)

Our focus on this angle is motivated by the fact that the role of shared preferences, compared to demonstrator competence, is relatively understudied in previous work on selective social learning. Additionally, we will consider non-deterministic social learning strategies akin to the Boltzmann policies for action selection, where each demonstrator agent is assigned a weight *w*^(*m*)^ that controls their probability of being selected as a social learning target:

$$\mathrm{Pr}\left(\text{learn}\;\text{from}\;\text{agent}\;m\right)\propto \exp\left(\frac{w^{\left(m\right)}}{\beta^{\text{ego}}}\right)$$ (7)

This simple rule describes the normative strategy that an agent seeking to maximise reward *should* use to learn socially in environments with preference variation, and will form the core of our rational models throughout the rest of the current work.

But to actually follow such a strategy, a social learner must either know or be able to infer the utility functions of the agents they encounter. Fortunately, there is considerable evidence that people are adept at inferring preferences from behaviour. Repacholi and Gopnik (1997) showed that children recognise by around 18 months that others can have preferences that differ from their own. Preschool-age children can go further, using statistical evidence in the form of deviations from random sampling in item choice to infer others’ preferences (Kushnir et al., 2010). Lucas et al. (2009, 2014) offer a rational model that provides a unified account of these (and other) results from the developmental literature. Drawing from work on modelling feature-based choice behaviour in econometrics, they cast preference estimation as a problem of Bayesian inference over choice models similar in form to 2. Subsequent work has validated this approach in more complex settings with adult participants (Jern et al., 2017). We do not offer a new model of how people infer preferences from choice behaviour; rather, our contribution is to apply this line of work to the problem of selective social learning. A rational learner, equipped with this kind of preference-inference machinery, can select social learning targets following Equations 6 and 7, with

$$w^{\left(m\right)}=𝔼\left[\text{sim}\left(U^{\text{ego}},U^{\left(m\right)}\right)|O^{\left(m\right)}\right]$$

$$p\left(U^{\left(m\right)}|O^{\left(m\right)}\right)\propto p\left(U^{\left(m\right)}\right)\prod_{\left(s,a\right)\in O^{\left(m\right)}}\pi^{\left(m\right)}\left(s,a|U^{\left(m\right)}\right)$$ (8)

where *π*^(*m*)^(*s*, *a*∣*U*^(*m*)^) is the Boltzmann-rational policy from Equation 4. In the interest of simplicity, we will model all learners as using the same neutral prior *p*(*U*^(*m*)^)—even though a more realistic model might include an egocentric bias in the form of a prior that skews towards the learner’s own preferences (Tarantola et al., 2017).

We will now test whether Equation 8 provides a good description of people’s *actual* social learning behaviour, across three experiments where participants make choices between hidden items in an environment with multiple potential demonstrators. In Experiment 1, we implement a setting where participants have observational access to all other agents’ choice behaviour, and so can evaluate preference similarity on a direct, individual basis. In Experiment 2, we consider the harder (but more realistic) setting where direct behavioural evidence is not available for all potential demonstrators, requiring participants to generalise based on visual cues to agents’ social group identity. Finally, Experiment 3 uses an extension of this setup to test people’s sensitivity to choice context in a setting where they align with different groups on different item features.

## EXPERIMENT 1: PREFERENCE INFERENCE AND SELECTIVE SOCIAL LEARNING

The aim of our first Experiment was to provide an initial test of people’s sensitivity to preference similarity in selecting social learning targets—i.e., can Equation 8 accurately predict how people behave in a simple social learning scenario? To this end, we set up a task in which participants had to make decisions under uncertainty with the opportunity to use social learning to more-effectively gather hidden rewarding items. The key idea behind the task design was to give people evidence indicative of other agents’ preferences via observations of their choices among items with observed features (after a participant has identified their own preferences with respect to these features). By forcing people to then choose between items with *hidden* features, but letting them first see which hidden item each of the other agents chose, we engineered a scenario where preference-based social learning was the only way for a learner to reliably gather rewards.

### Methods

#### Participants.

We recruited 150 UK-based adults via the online platform Prolific. Mean age was 36.2 (*SD* 11.1), 62 participants were male, 78 female (10 selected ‘prefer not to say’). Participants were reimbursed for their time with a base payment of £1.05 (based on an estimated rate of £9.00/hr). To incentivise performance, we awarded an additional bonus payment of £0.01 for every 5 points scored (mean total reward £1.34, min £1.15, max £1.43). The mean experiment duration was 7 m 55 s (*SD* 4 m 27 s).

#### Design and Procedure.

**Figure 2.**
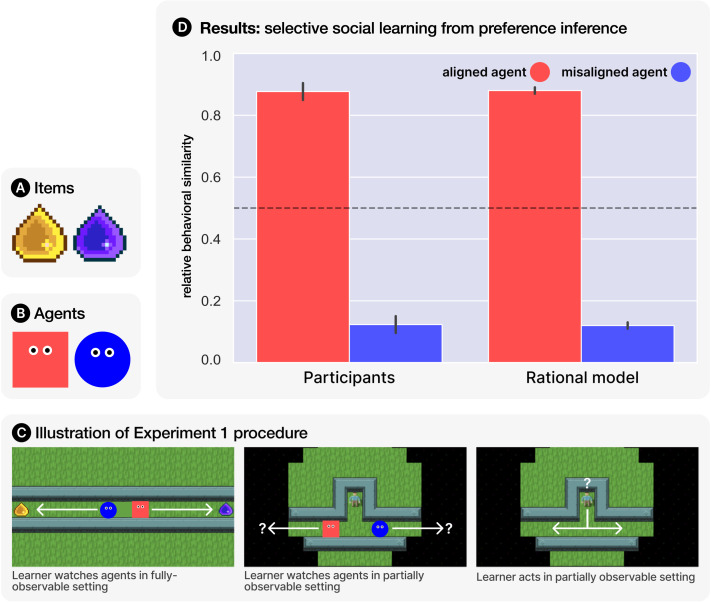
Experiment 1. **(A)** The two different items in the environment. **(B)** The two simulated agents observed by participants. **(C)** A simplified illustration of the three stages that each participant experienced. **(D)** A comparison of participants’ recorded social learning behaviour to the rational preference-inference-based strategy given in Equation 8. Bar heights measure the *relative selectivity* with respect to the aligned vs misaligned agent, based on the similarity between trajectories in 2D space. Error bars give 95% confidence intervals, and the dashed line at 0.5 indicates a baseline of no selectivity (i.e., indiscriminate social learning or asocial random choice).

In the task, participants controlled an avatar in a virtual 2D gridworld environment^3^ created using the GriddlyJS framework (Bamford et al., 2022), where their goal was to earn points by collecting gems (Figure 2A). Gems were of two different colours (yellow and purple) and participants’ utility functions were set such that they earned more points for one colour than the other (randomly assigned), with explicit instructions given at the start of the experiment how many points they would earn for each gem colour. Utility functions also included a fixed cost of one point lost per step taken in the environment, incentivising efficient navigation and gem collection. In addition to the gems, the gridworld environment also contained two simulated agents, rendered as stylised geometric avatars (Figure 2B). Participants observed each agent execute 2 trajectories in a fully-observable version of the environment, where one agent consistently chose the yellow gem and one agent the purple gem. Participants then had to navigate a *partially observable* version of the environment. Here they again had to choose between two possible paths, but crucially could not see which path led to which gem. Before making their choice, participants observed the path chosen by each of the two simulated agents. An illustration of this procedure is given in Figure 2C. If participants followed the rational selective social learning strategy outlined above, then we would expect them to choose the same hidden path as the agent that they previously inferred shared their gem preference. Each participant observed a total of 8 trajectories (4 per agent) and made a total of 4 path choices (of which we analyse only the 2 made in the partially observable layout). There were no between-subject conditions, and both gem preference and agent presentation order were counterbalanced.

### Results

To measure participants’ selection of social learning targets, we considered the similarity between their recorded behaviour and that of both simulated agents. Given a pair of trajectories in 2D space (*τ*_*i*_, *τ*_*j*_), we define the similarity *s* ∈ (0, 1] as

$$s\left(\tau_{i},\tau_{j}\right)=e^{-F\left(\tau_{i},\tau_{j}\right)}$$ (9)

where *F* is the discrete Fréchet distance, which gives a robust measure of the distance between two paths of possibly varying length (Eiter & Mannila, 1994). We use this to compute the mean behavioural similarity with respect to each of the two potential social learning targets ($\bar{s}$_1_, $\bar{s}$_2_). If participants learned from the two agents indiscriminately (or chose asocially by picking a path at random), we would expect $\bar{s}$_1_ ≈ $\bar{s}$_2_. We can therefore take the value $\frac{{\bar{s}}_{m}}{{\bar{s}}_{1}+{\bar{s}}_{2}}$ as a measure of *relative selectivity* in favour of agent *m*. Figure 2D shows a comparison of participants’ social learning behaviour to that predicted by the model given in Equation 8 (using *β* = 0.2). Bars measure the degree of selectivity for the ‘aligned’ (same gem preference) over ‘misaligned’ (opposite gem preference) agent. As predicted by the model, participants were overwhelmingly selective in learning from the path choices of the agent who shared their utility function over gems (mean relative similarity 87.5%). In addition to the visual analysis in Figure 2, we conducted a two-tailed Binomial test with a null hypothesis of indiscriminate social learning, i.e., Pr(copy aligned agent) = Pr (copy misaligned agent) = 0.5, which yielded *p* < 0.001 with *k*/*n* = 126/144 trajectories. We can therefore conclude that when behavioural evidence of agents’ preferences was available, people’s choice of who to copy was well-captured by a selective social learning model that infers these preferences and then uses the inferences to weight a distribution over potential demonstrators.

### Discussion

While a useful starting point, dyadic comparison based on direct inference of individuals’ utility functions has some significant shortcomings. First, we assumed access to direct observations of choice behaviour from any agent that a social learner might wish to learn from, which seems unrealistic. Second, even if we can assume observational familiarity, inferring an agent’s utility function can be a costly process in more complex environments; moreover, there is typically not enough time to sample everyone’s behaviour sufficiently to form a detailed model of them from scratch. But more fundamentally, the assumption that each agent’s utilities are independent of all the other agents in the environment or broader population is implausible. Values and preferences are not formed in a vacuum, and are often correlated across different individuals. Within a given population, knowledge of one agent’s preferences may indeed tell you something about the preferences of another, since the foundations that shape these preferences, whether evolutionary or cultural, are to some extent shared. A successful strategy for selecting social learning targets ought therefore to take this into account, and consider agents not only as individuals, but also as elements within a larger social structure.

In the remaining sections of the current work, we develop our account from the individual level to focus on social groups. The core of our model will remain unchanged; i.e., assign weights to potential demonstrators based on expected preference similarity, and then use these weights to guide learning behaviour following Equation 7. The difference will be in how this expectation is computed, and what kind of evidence the model considers.

## CATEGORIZATION AND SOCIAL GROUPS

Before outlining our group-level model, a brief discussion of social categorisation and group-centric representations is in order. Categorization is of course a central feature of human cognition, with categories serving as ‘cognitive economizers’ across many different domains, allowing people to reason about the properties of individual members of a category by drawing on their cumulative knowledge or experience of the category as a whole (Markman, 1989; Nisbett et al., 1983; Osherson et al., 1990). Social cognition is no exception: as well as the tendency to draw inferences about others’ desires and motivations from observations of their behaviour, we also tend to categorise others as belonging to groups or types (Fiske & Neuberg, 1990; Liberman et al., 2017; Macrae & Bodenhausen, 2000). While this can lead us astray, such as when we overgeneralise stereotyped beliefs to form unfair judgements of individuals, it is also a powerful tool for making sense of our complex social environments (Devine, 1989; Fiske & Neuberg, 1989, 1990; Tajfel et al., 1971). There is some debate as to how people use social categories to make inferences and predictions about individuals, as well as how this changes from childhood to adulthood. It is suggested that social categories may be used more for predicting interactions between individuals and prescriptive or deontic judgements (what people *ought* to do), than for predicting psychological properties such as goals, values or beliefs (Foster-Hanson et al., 2021; Kalish & Lawson, 2008; Shutts et al., 2013). Nevertheless, there is evidence that people associate values and preferences with social category assignments, expecting preferences to be more similar within social categories than across them. Monolingual infants generalise food preferences across same-language speakers but not different-language speakers (Liberman et al., 2016), and five-year-old children expect an unfamiliar child to prefer the same activity as another child from their ethnic group, rather than a second activity preferred by a child from a different ethnic group (Diesendruck & HaLevi, 2006). These findings suggests that social learners might be able to select demonstrators with aligned preferences by making inferences from social category information—in essence an extension of the rational strategy given in Equation 8 to the (more general) case where direct evidence of demonstrators’ choice behaviour is unavailable.

However, there is a simpler way that cues to group identity could guide selective social learning, without any rational inference about agents’ preferences. While social categorization might arise from mechanisms similar to those in other categorization domains, there is a key difference: people can identify *themselves* as members of particular social categories, considering those who share their category membership as members of their ‘ingroup’, and those who do not as ‘outgroup’ (Baron & Dunham, 2015; Dunham, 2018; Meltzoff, 2007). This simple distinction exerts significant influence on social attitudes and behaviour: people consistently demonstrate a preference towards ingroup members, such as in choice of friends, distribution of rewards and penalties, or receptivity to valenced information about group members (Gramzow et al., 2001; Greenwald et al., 2002; Tajfel et al., 1971). Indeed, there is a good deal of evidence that children prefer to learn from members of their ingroup, along dimensions of native language or accent (Buttelmann et al., 2013; Kinzler et al., 2007, 2011; Lev-Ari & Keysar, 2010), as well as sex (Frazier et al., 2012; Ma & Woolley, 2013; Shutts et al., 2010; Taylor, 2013).

We might therefore conclude that simple ingroup biases cover the full role of group labels in selective social learning. But as we suggested in the introduction, people sometimes learn preferentially from *outgroup* members, such as children preferring to learn from teachers or other adults rather than from their same-age peers (Abramovitch et al., 1980; Jaswal & Neely, 2006; Rakoczy et al., 2010; Seehagen & Herbert, 2011; Wood et al., 2012). In these cases, the child does not favour the adult because they believe that *they* are also an adult, but presumably because members of the ‘adult’ category are more suitable to learn from due to their greater experience or expertise. In a recent paper, Cunningham et al. (2023) presented evidence of a developmental shift in the influence of demonstrator gender on social learning decisions. While adolescent boys demonstrated significant own-gender bias in choosing whom to learn from, adults’ selections aligned more with western cultural stereotypes regarding gender differences in domain-specific competence. As in the example above, this is evidence of group-directed social learning decisions based not on ingroup bias but on beliefs about the relative *suitability* of different groups as social learning targets (even when those beliefs may be incorrect and reflect harmful cultural stereotypes, as in this case).

However, categories such as ‘teacher’, ‘adult’, ‘native English speaker’, or ‘male’, while diverse in their defining properties and the predictions they might license about members, are similar in two key senses: *stability* and *cultural significance*. An individual’s native language will persist across contexts or environments, and a teacher outside of the classroom is still a teacher. It seems that a full account of group-based selective social learning ought to be able to deal with much more ephemeral or contingent social categories, that may be meaningful only in specific contexts and are not generally given broader cultural significance. A *rational* social learner, seeking to select a social learning target on the basis of group affiliation, should not blindly follow a naïve preference for ingroup over outgroup, nor a broad cultural stereotype; rather they should draw on prior evidence or understanding about the significance of different group identities within the current task or environment context.

In the following section, we formalise both of these approaches as they relate to our focus on navigating preference variation. We then present the results of two further Experiments that aim to distinguish between them in human social learning.

## SELECTIVE SOCIAL LEARNING VIA GROUP IDENTITY

### Naïve Ingroup Bias

The most basic form of group-based selective social learning is what we will call the *naïve ingroup bias*; i.e., learn from agent *m* only if agent *m* is a member of my ingroup (along some salient dimension). A version of this strategy can be expressed using the general agent selection rule introduced in Equation 7, with weights given by

$$w^{\left(m\right)}=\left\{\begin{array}{ll} 1 & \text{if}\;z^{\left(m\right)}=z^{\text{ego}}\\ -1 & \text{if}\;z^{\left(m\right)}\neq z^{\text{ego}} \end{array}\right.$$ (10)

where *z*^(*m*)^ denotes the group assignment of agent *m*. We can view this model as a generalisation of the more specific ingroup social learning biases discussed in the previous section, from work such as Buttelmann et al. (2013), Frazier et al. (2012), Kinzler et al. (2007, 2011), Lev-Ari and Keysar (2010), Ma and Woolley (2013), Shutts et al. (2010), and Taylor (2013). As we suggested, and as evidenced by Cunningham et al. (2023), this kind of simple bias cannot capture the full role of group-level representations in selective social learning. In particular, we can ask how social groups might play into the rational preference-based account outlined in Optimal Selective Social Learning section. We discussed in Experiment 1: Preference Inference and Selective Social Learning section how the distribution over agents’ preferences be inferred from direct observations of agents’ choice behaviour (S2 in Table 1). But even before we have the opportunity to observe a particular agent’s choices we may have some strong inductive biases what their utility function might be. That is, similar to Rawls’ ‘thin conception of the good’ (Rawls, 1971), there are certain values or preferences—such as avoiding pain or seeking food when hungry—that are near-universal to the human experience. This is not to say that individuals cannot still vary substantially in the things that they value or pursue (the richer ‘full conception’); but simply that through some combination of evolutionary and cultural forces there exists a degree of ‘conservation’ between individuals’ utility functions, such that we are able to say *something* about the likely preferences of an arbitrary decision-maker even while knowing nothing about them.

**Table 1.** Hypothesised strategies for preference-based selective social learning

Strategy	Description	Evidence used	Experiments
S1: *Indiscriminate*	No systematic favouring of any one agent over any other; can capture either indiscriminate social learning or asocial random choice.	None	1, 2
S2: *Rational dyadic*	Rational selective social learning *only* on the basis of direct/dyadic *behavioural* evidence. Indiscriminate when this evidence is unavailable.	Behaviour	1, 2
S3: *Naïve cue-based*	Selective social learning from agents that share your explicit group cue, with no regard for the significance of these cues. Indiscriminate when explicit cues are not observable.	Explicit cues	2, 3
S4: *Rational cue-based*	Use both direct behavioural evidence and explicit cues to inform a rational selective social learning strategy, depending on the relative availability of each (indiscriminate if neither available).	Both	2, 3
S5: *Agreement frequency*	Selective social learning from agents whose group cue matches the group with which you’ve agreed most frequently over all past choices. Note that while this approach does use both behavioural evidence and group cues, it does not involve any actual inference of preference distributions.	Both	3

And what if, instead of an entirely unknown individual, you were asked to predict something about someone’s preferences knowing the different social groups to which they belong? For some combinations of grouping and choice context, you would find yourself able to make stronger predictions, even before observing any actual choice behaviour. That is, we can say that the prior over utility functions conditioned on group identity *p*(*U*^(*m*)^∣*z*^(*m*)^) is in general less flat (i.e., more informative) than the generic prior *p*(*U*). Partly this is explained by the fact that some social groups are formed specifically around a particular set of preferences or values, such that sharing those preferences or values is the defining characteristic of that group’s members—for example, musical subcultures, hobbyist groups, or activist organisations. But in addition to this, people may be motivated to align themselves with the other members of their social groups. This means that even groups which are not constructed around similar values may tend over time towards greater similarity in the values or preferences of their members.

So, our argument is that knowledge of agents’ group identities can be used to reduce the uncertainty over their unknown utility functions. But what does it mean to have knowledge of agents’ group identities? We suggested earlier that the way agents can be meaningfully organised into groups will depend on the context; i.e., there is not some single ‘ground-truth‘ social group structure that we assume our learner has access to. Instead, people will often express *explicit cues* that convey something about their social identity. We can treat these cues as a noisy signal *ϕ* of some underlying latent social identity *z*. If we then consider agents as making group-level decisions on the basis of *ϕ* rather than *z*, our naÏve ingroup bias model (S3 in Table 1) takes a form such as

$$w^{\left(m\right)}=-d_{\phi }\left(\phi^{\left(m\right)},\phi^{\text{ego}}\right)$$ (11)

where *d*_*ϕ*_ is some measure of the distance between agents’ explicit cues.

### Rational Group-Based Selection

In contrast to this simple strategy, we propose a rational account under which people learn about the *significance* of explicit social cues. That is, people learn over time a relationship between explicit cues, the latent groups they signal, and the typical attributes (such as utility functions) of members of those groups. Thus, when faced with an unknown individual *m* expressing explicit cue *ϕ*^(*m*)^, an observer can draw upon past experience and use the cue to condition their predictions about the individual; e.g., they use *p*(*U*^(*m*)^∣*ϕ*^(*m*)^) rather than simply *p*(*U*^(*m*)^).

**Figure 3.**
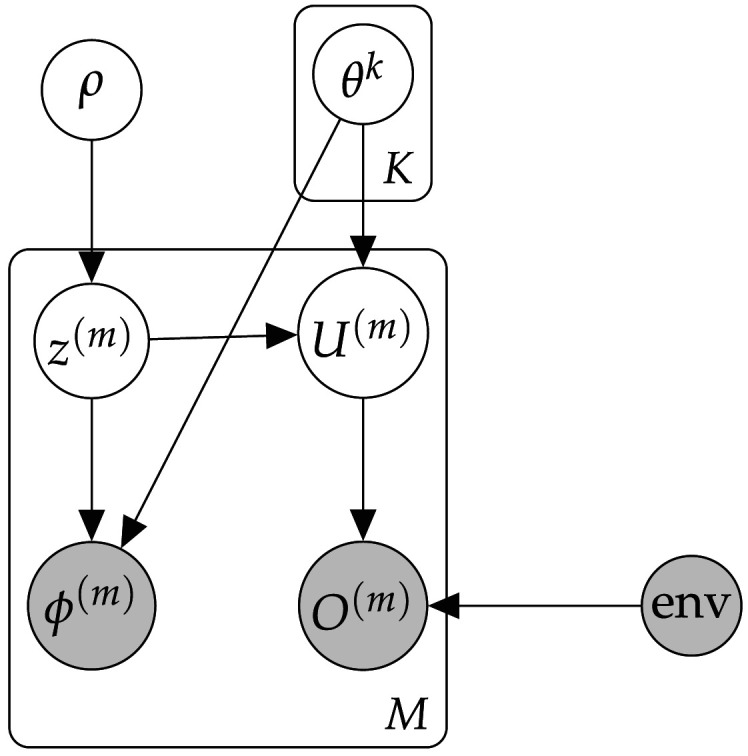
Graphical illustration of our generative group-based model. Here, *K* is the true number of latent groups and *M* is the total number of agents. *ϕ* (agents’ explicit cues), *O* (their choice behaviour) and ‘env’ (the choice environment—in our experiments the virtual item-containing gridworld) are observable; all other variables are hidden.

To formalise this proposal, we use a similar approach to Gershman et al. (2017) and Lau et al. (2020), with the key difference that they consider latent group inference strictly in the *absence* of explicit group labels, whereas we consider it through the lens of identifying the significance of such labels. First, we assume that a given population of agents is partitioned into some number of non-overlapping latent groups. The latent group membership *z*^(*m*)^ of each individual agent *m* then strongly influences (though doesn’t fully determine) *both* their utility function *u*^(*m*)^ and explicit cue *ϕ*^(*m*)^. We can express this using a Dirichlet Process Mixture Model (DPMM), a Bayesian non-parametric model with a history of use in capturing categorisation and grouping phenomena in cognitive science (Gershman & Blei, 2012; Griffiths et al., 2007; Sanborn et al., 2010; Zhao et al., 2022), including modelling social groups in the context of choice behaviour (Gershman et al., 2017). Expressed as a DPMM, our generative model (also illustrated in Figure 3) is given by

$$\theta^{k}∼G_{0}$$

$$z^{\left(m\right)}∼\text{Categorical}\left(\rho \right)$$

$$U^{\left(m\right)}∼p\left(U^{\left(m\right)}|\theta^{z^{\left(m\right)}}\right)$$

$$\phi^{\left(m\right)}∼p\left(\phi^{\left(m\right)}|\theta^{z^{\left(m\right)}}\right)$$

$$O^{\left(m\right)}∼p\left(O^{\left(m\right)}|U^{\left(m\right)},\text{environment}\right)$$ (12)

Unpacking this, we have each agent *m* assigned to a single latent group *z*^(*m*)^ sampled from a categorical distribution with mixture weights *ρ*. Each group *k* has an associated set of parameters *θ*^*k*^ (drawn from a base distribution *G*_0_); each agent’s utility function and explicit group cue are both then drawn from distributions parameterised by their group’s *θ*. Finally, each agent’s utility function will combine with the environment to produce the actions we observe them taking (*O*^(*m*)^). The mixture weights *ρ* are generated via a 'stick-breaking process' (Teh, 2010), that is

$$\beta_{c}∼\text{Beta}\left(1,\alpha \right),\;c=1,2,3,\ldots ,$$

$$\rho_{c}=\beta_{c}\prod_{j=1}^{c-1}\left(1-\beta_{j}\right)$$ (13)

One major advantage of representing the social value surface over feature dimensions with a DPMM rather than a finite Gaussian mixture model is that it allows a theoretically infinite number of clusters (groups) to be represented. This is attractive for settings such as ours, in which we do not want to grant an observer prior knowledge of the ‘true’ number of groups or categories present in the environment. The stick-breaking process can be seen as expressing a ‘rich-get-richer’ inductive bias, where a new datapoint (in our case, agent) is assigned to an existing group with probability proportional to that group’s current size. This inductive bias is appropriate—when partitioning agents into groups, we typically want to consider a small number of populous groups as more likely than a large number of relatively uninhabited groups, other things being equal.

If a rational social learner, observing the choice behaviour {*O*^(*m*)^} and explicit cues {*ϕ*^(*m*)^}, can invert this hierarchical model to infer the group parameters {*θ*^*k*^}, then they can use these to obtain the conditional distributions *p*(*U*^(*m*)^∣*ϕ*^(*m*)^) by marginalising over potential latent group assignments as

$$\begin{array}{l} p\left(U^{\left(m\right)}|\phi^{\left(m\right)}\right)\propto \sum_{z^{\left(m\right)}}p\left(U^{\left(m\right)}|z^{\left(m\right)}\right)p\left(z^{\left(m\right)}|\phi^{\left(m\right)}\right)\\ \;=\sum_{z^{\left(m\right)}}p\left(U^{\left(m\right)}|\theta^{z^{\left(m\right)}}\right)\frac{p\left(\phi^{\left(m\right)}|\theta^{z^{\left(m\right)}}\right)p\left(z^{\left(m\right)}\right)}{p\left(\phi^{\left(m\right)}\right)} \end{array}$$ (14)

and then can use this to weight unfamiliar agents by

$$w^{\left(m\right)}=𝔼\left[\text{sim}\left(U^{\text{ego}},U^{\left(m\right)}\right)|\phi^{\left(m\right)}\right]$$ (15)

This is the ‘rational cue-based’ strategy denoted as S4 in Table 1. In settings where agents vary only by utility function (so have equal competence *β*), this is the optimal approach to selecting social learning targets on the basis of explicit cues to group identity. That is, this is what a social learning agent *should* do in order to maximise their expected reward. But we are interested here in the question of which model better captures people’s actual social learning choices—which of the strategies in Table 1 do people follow, when given access to either direct behavioural evidence or explicit cues to group identity?

## EXPERIMENT 2: TESTING THE USE OF GROUP INFORMATION IN SELECTIVE SOCIAL LEARNING

To answer this question, we conducted a second online experiment using a new custom virtual environment. This experiment can be seen as an extension of Experiment 1, using the same core mechanic but introducing a simple group structure and a richer preference space, in order to test the two models laid out in the previous section.

**Figure 4.**
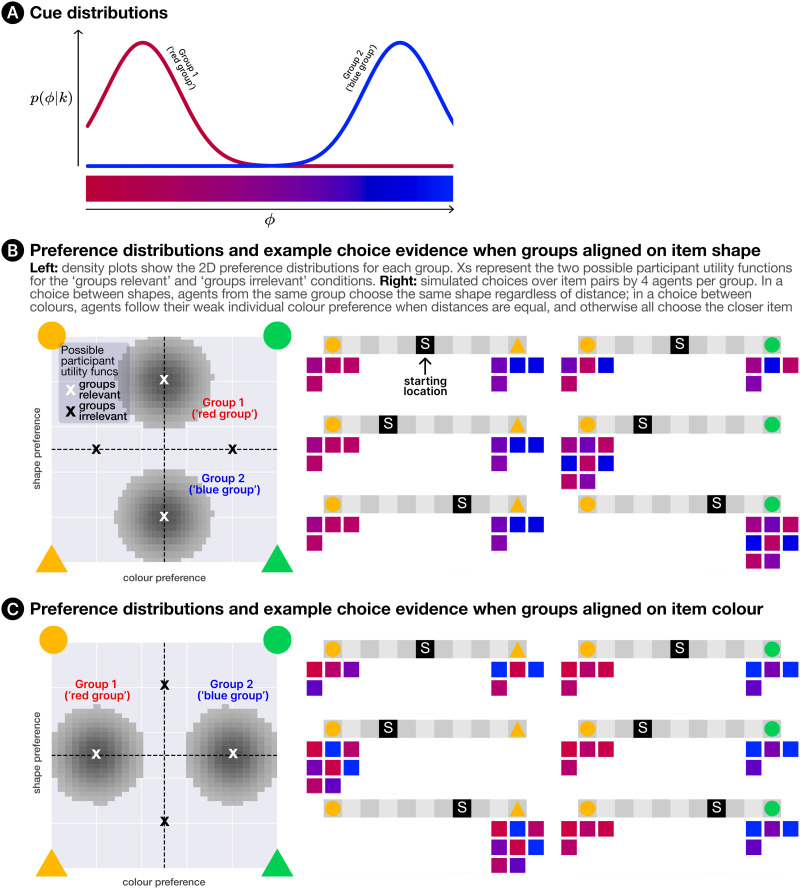
An illustration of the group setup for Experiment 2. (A) shows the *ϕ* distributions for each group. (B) and (C) show the utility function distributions when group identity is associated with shape preference or colour preference respectively. Regardless of which dimension group membership is correlated with, there is a degree of intra-group variation for both preferences. Marked with Xs are the possible *participant* utility functions (in white for the ‘groups relevant’ condition and in black for the ‘groups irrelevant’ condition). Finally, for each group alignment we also illustrate some example choice evidence, where four agents sampled from each group made choices between different item pairs from different starting locations.

Through action and observation, participants first learned about their own preferences, and were given the opportunity to learn about the preferences of the broader agent population. Participants were then presented with scenarios where, as in Experiment 1, they faced a choice between two social learning targets, with the aim of maximising reward on a simple decision task. The key difference between Experiments 1 and 2 is in the evidence that participants had available when selecting social learning targets. In Experiment 1, there was a single source of information about other agents’ properties—their choice behaviour—which participants had access to for all agents in the environment (and would use to infer their utility functions). In Experiment 2, there were two types of evidence. Direct observations of behaviour were still available for *some* (though not all) agents; but in addition, some agents displayed a visual cue to their social group membership (*ϕ*^(*m*)^, as described in Selective social learning via group identity section) in the form of their avatar’s colour, which was sampled from a perceptually uniform red-blue spectrum^4^. Agents were, in fact, drawn from one of two non-overlapping groups, with one group expressing colours closer to the red end of the spectrum, and the other closer to the blue end (see Figure 4). Membership of these two groups was also noisily associated with different item preferences—but cue shade and preferences were not directly related themselves; e.g., the most red agent in the red group did not necessarily have stronger preferences than a moderately red agent from the same group. Note that participants were not explicitly informed about these groups at any point, and were instead left to infer their existence from the behaviour and features of individual agents.

Table 1 enumerates the possible social learning strategies that a participant could follow based on these two types of evidence. The *Indiscriminate* strategy is included for completeness, although given our findings from Experiment 1 we expected that participants would *at least* be able to select a social learning target on the basis of direct behavioural evidence of similar preferences. The more interesting question, and the specific focus of this experiment, involves testing strategies 3 and 4 against one another. Is the role of social group information limited to supporting a naïve ingroup bias (Equation 11)? Or, can it be used for rational preference-based selective social learning (Equation 15), even in the absence of an explicit ingroup/outgroup distinction from the social learner’s perspective?

### Methods

#### Participants.

We recruited a new set of 150 adult UK-based participants from Prolific. Mean age was 36.2 (*SD* 12.2); 60 participants were male and 89 female (1 selected ‘prefer not to say’). Participants were reimbursed for their time with a base payment of £1.50 (based on an estimated rate of £9/hr). To incentivise performance, we awarded an additional bonus payment of £0.01 for every 3 points scored (mean total reward £1.87, min £1.60, max £1.94). Mean experiment duration was 9 m 29 s (*SD* 3 m 38 s).

#### Design.

We manipulated two factors between participants. Factor 1 was the *relevance* (to the participant) of the two explicit social groups. That is, the simulated agents’ group identities were correlated with their utility functions along *only one* of the two item dimensions (with Figure 4 illustrating the preference distributions for both possible alignments). Similarly, the participant was assigned a utility function that varied only along one feature dimension. By manipulating whether or not these dimensions matched, we controlled whether the latent groups (and thus the explicit cues) were informative or irrelevant to the selection of suitable social learning targets. A second factor concerned the assignment of participants’ own explicit group cues, which could be either *invisible* (participant sees their avatar as grey), *arbitrary* (matched to the mean value of either group’s *ϕ* distribution at random), *matched* (matched to the mean value for the group aligned with their utility function) or *mismatched* (matched to the mean value for the group misaligned with their utility function). We used a partially-crossed design, since the possible ways of assigning a participant’s explicit cue depends on the relationship between cues and utility functions^5^. Table 2 shows the five factor combinations that were used (numbered), along with the three omitted (marked with crosses). Participants were split evenly across these five conditions (30 per condition). We counterbalanced participant item preferences, group-preference alignment and the order of both agent observations and starting locations.

**Table 2.** The five (between-participant) conditions tested in Experiment 2

	Cue invisible	Cue arbitrary	Cue matched	Cue mismatched
Groups irrelevant	1	2	x	x
Groups relevant	3	x	4	5

#### Procedure.

Participants in Experiment 2 were tasked with controlling an avatar through different layouts of a 2D gridworld environment, which was again implemented using GriddlyJS (Bamford et al., 2022). There were 12 layouts in total (8 in Phase 1, 2 in each of Phases 2–3), with each containing a subset of four possible items. Items varied along two dimensions: colour and shape. As in Experiment 1, points were earned by navigating to and ‘collecting’ items, and the number of points earned for each type of item was determined by the participant’s utility function. Crucially, these utility functions were set such that, for a given participant, the reward for a given item depended on only *one* of the two dimensions. For instance, a participant might earn more points for yellow items than green, but earn the same points for circles as triangles (of the same colour). In addition to the participant’s avatar, the environment also contained a population of virtual agents, which were organised into two latent social groups and engaged in item collection to maximise their own rewards. Due to the intragroup variance inherent in the generative model (Equation 12), agents had (in general) a preference along both feature dimensions—but had a dominant preference determined by their group identity. Each agent’s group identity also parameterised the Gaussian distribution from which their explicit cue (*ϕ*) was sampled. The group distributions for both preferences and cues are illustrated in Figure 4. As in Experiment 1, participants and simulated agents incurred a cost of 1 point for each step taken in the environment, incentivising efficient item collection.

The experiment was divided into three phases, with the procedure being the same for all participants. **Phase 1:** in the ‘exploration phase’, participants were alone in the virtual environment, and engaged in exploration to learn about their *own* utility functions by collecting different items and observing the resulting payoffs (with all item features clearly visible). **Phase 2:** in the ‘known agents’ phase, participants were introduced to two agents (“Alex” and “Bob”), whom they first watched make their own choices between fully visible items in the environment (the ‘evidence levels’). Participants then faced a series of choices (the ‘test levels’) between items with *hidden* features (rendered as mystery boxes), after first observing which hidden item each of Alex and Bob travelled to. Either Alex or Bob had the same feature preferences as the participant (with the other having opposite preferences). Alex and Bob also did not exhibit group cues *ϕ*, instead showing up as grey. Phase 2 was therefore a replication of Experiment 1. **Phase 3:** the ‘unknown agents’ phase the same basic structure as phase 2. The differences were that in this phase all the virtual agents *did* exhibit group cues *ϕ* (showing up as a colour on the red-blue spectrum from Figure 4) and crucially, the potential demonstrators during the hidden-item test levels were *not* the same agents participants saw in the visible-item evidence levels. That is, during the evidence levels, participants saw two agents from each group (“Charlie”, “David”, “Eric” and “Frank”) choose between the different items over a number of trials^6^. Then in the test levels, participants saw which mystery boxes were chosen by one *new* agent from each group (“George” and “Henry”). To make a rational choice of demonstrator during the test levels, a participant would therefore have to generalise on the basis of the group cues *ϕ* the evidence obtained for the previous four agents—rather than treating all agents as individuals unrelated to one another by any latent social structure.

### Results

**Figure 5.**
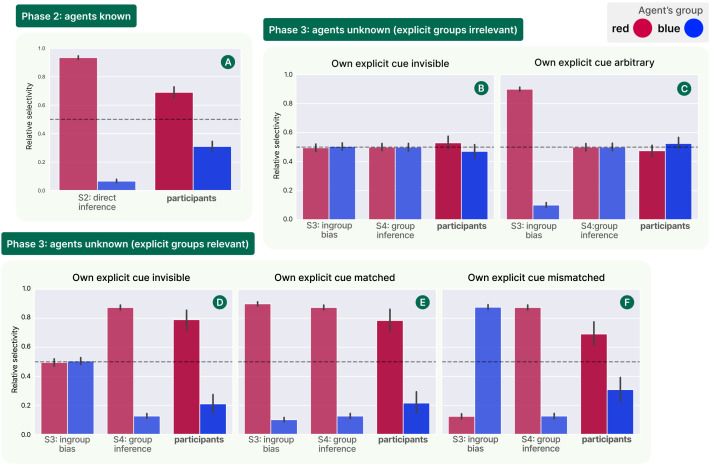
Results of Experiment 2. As in Experiment 1, bar heights measure relative selectivity in favour of each of two available demonstrators, and error bars give 95% confidence intervals. Here, each demonstrator represents a different social group. For the ‘known agents’ phase (phase 2), we compare participant data against the individual inference model used in Experiment 1. For the ‘unknown agents’ phase, we compare against the ingroup bias model (Equation 11 with *d*_*ϕ*_ as the normalised distance between agent colours in CIELAB space) and the rational group inference model (Equation 15). Across the 5 conditions, participants’ social learning behaviour is best captured by the rational model.

Participants’ behaviour in Experiment 2 was analysed following the same procedure as in Experiment 1 (described in Experiment 1: Preference Inference and Selective Social Learning section). Participants were counterbalanced in their assigned utility function and explicit cue, but for the purposes of analysis and visualisation we collapse this counterbalancing and visualise participants as either belonging to the ‘red‘ group (where group preferences are relevant) or arbitrarily labelled as such (where they’re not). Figure 5 shows the results of this analysis, for both the known and unknown agents phases (and all conditions). The bar heights measure relative selectivity in favour of the potential social learning targets from each group, with a dotted reference line marked at 0.5—as in Experiment 1, we also used a two-tailed Binomial test to assess the significance of participants’ departures from this baseline.

Looking first at the results for the known agents phase 2 (Panel A), we replicate the findings from Experiment 1—that is, given direct observation of both demonstrators’ choice behaviour, participants are strongly selective (0.690 (414/600 trajectories), *p* < 0.001) in favour of the agent whose utility function they could infer as being more similar to their own. The remaining two panels address the question at the heart of this experiment: how do people imitate selectively on the basis of explicit group cues instead of direct behavioural evidence? When these cues *did not* carry relevant (to the participant) information about members’ utility functions (Panels B–C), participants showed no significant selectivity (B: relative selectivity 0.525 (63/120), *p* = 0.523; C: 0.475 (57/120), *p* = 0.648) in favour of either group, instead either employing indiscriminate social learning or (asocial) random choice. Importantly, this was true even when the participant was themselves labelled with an arbitrary explicit group cue—suggesting that participants rejected the naÏve ingroup bias strategy based on an understanding that it would not be effective. When cues *did* carry relevant information (Panels D–F), participants were able to recognise this and favoured learning from the agent whose group was aligned with the participant’s own utility function (D: relative selectivity 0.790 (98/124), *p* < 0.001; E: 0.784 (91/116), *p* < 0.001; F: 0.692 (83/120), *p* < 0.001). Most notably, this result held not only when the participant had no explicit group label of their own, but even when the participant’s explicit cue was actively *misleading*. That is, participants correctly inferred the red group to contain more suitable social learning targets even when they themselves were marked as though they belonged to the blue group. We do note that the effect is slightly weaker in this condition (relative to the invisible and matched conditions), suggesting that some participants may have been reluctant to go against their perceived group identity. However, the overall trend is still robust, and we believe that this result in particular provides strong evidence for our rational group inference account against a more naÏve strategy based on explicit ingroup bias.

To more clearly illustrate the implications of these results for the different possible strategies given in Table 1, we conducted a model comparison. Each model was provided with evidence equivalent to that seen by participants for each condition, and used this to compute weights over the two potential social learning targets. These weights were then used to simulate stochastic selections of social learning targets following Equation 7 (with a constant *β* = 0.5 used for all models). For the known agents phase 1 (Panel A), behavioural results were compared against the same individual-level preference inference model used in Experiment 1 (S2 in Table 1)—again we see that participants’ behaviour is well-captured by the model. For the unknown agents phase, we implemented additional models based on the two explicit-group-cue-based strategies outlined in Equations 11 (‘ingroup bias’) and 15 (‘group inference’). Over all conditions (Panels B–F), the experimental data are best fit by the rational group inference model (S4 in Table 1). Specifically, while the ingroup bias model predicts the behavioural data in condition E, it fails for all others, where the participant’s own group label is either not available or contradicts their ‘true’ latent group membership, or explicit group cues are irrelevant.

As an additional check, we also analyse whether participants’ behaviour can be better accounted for by simpler choice heuristics that don’t use any evidence of agents’ preferences or group identity:

- *The ‘alternating’ strategy*: picks the direction opposite to whichever direction was picked last
- *The ‘closest’ strategy*: picks whichever item is closest, or chooses randomly if items are equidistant
- *The ‘copy first’ strategy*: imitates (at each level) the path choice of the first agent observed
- *The ‘copy last’ strategy*: imitates (at each level) the path choice of the most recent agent observed

**Figure 6.**
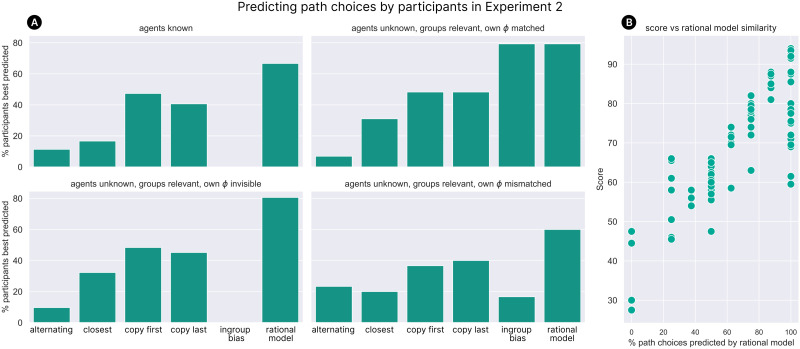
(A) The percentage of participants in Experiment 2 best fit by each of various strategies (where a participant is considered to be best fit by a strategy if that strategy correctly predicts the highest proportion of their path choices). Since our aim is to compare our rational account to simpler alternatives, we exclude the ‘groups irrelevant’ conditions, where the rational model makes no particular predictions. (B) The relationship between how well each participant’s behaviour was predicted by our rational model, and the score they achieved on the test levels of the task.

For each participant, we use these strategies (in addition to the ingroup bias and rational models) to predict their individual path choices across the hidden-item test levels. For each strategy, we then count the number of participants for whom that strategy correctly predicts the highest proportion of path choices^7^. Figure 6A shows that across all conditions, the majority of participants are best fit by our preference-based rational model—and in general, participants’ behaviour cannot be accounted for using simpler heuristics such as ‘alternate path choices’ or ‘choose the closest item’. Finally, to determine whether similarity to our rational model corresponds with better task performance (as we would expect), we compare participants’ scores during the test levels against the percentage of their path choices correctly predicted by the rational model. Figure 6B shows that there is a strong positive relationship, confirming that conformity to the rational model was indeed associated with better task performance.

This experiment demonstrates that people can make rational use of group information for selective social learning, and are not limited to following a naïve ingroup bias. Of course, while social group identity can be a useful guide to selecting social learning targets, we don’t generally align with the same group in every decision-making context. But for Experiment 2, in conditions where the group preference distributions were relevant to the participant, the ‘correct’ choice of which group to learn from was the same throughout the experiment. Our results from Experiment 2 therefore permit a slightly simpler alternative account, in which the social learner still uses both group cues and behavioural evidence but simply tracks over time which group they tend to agree with more (rather than actually recovering the underlying distributions). To properly distinguish between this simpler hypothesis (included as S5 in Table 1) and our rational group-based model (S4), we conduct a final experiment to test whether participants can selectively learn from agents in *both* of two different groups, depending on the presented choice context.

## EXPERIMENT 3: CONTEXT-SENSITIVITY IN GROUP-BASED SOCIAL LEARNING

Experiment 3 has a similar basic setup and structure to Experiment 2, but where group membership in Experiment 2 was associated with an individual’s preference along a *single* feature dimension (i.e., colour or shape) we now have group membership associated with *both* preference dimensions. For a more challenging test of selective social learning, participants’ utility functions are then assigned such that they align with Group 1 along one dimension but with Group 2 along the other. During the ‘test phase’, each participant has to choose in separate tests between items that differ only in colour, and items that differ only in shape. Crucially, we inform participants before each choice trial which of the two feature dimensions are relevant (while the actual features of each item are still obscured, as in the previous Experiment). A rational social learner, having inferred the preference distributions associated with each group, should be able to select different social learning targets depending on which feature is relevant in the context of a given choice problem. In contrast, a learner following either a naïve ingroup bias or the agreement-frequency strategy would *always* imitate agents from the same group regardless of context.

### Methods

#### Participants.

We recruited a further 60 adult UK-based participants from Prolific. Mean age was 39.1 (*SD* 13.1); 30 participants were male and 29 female (1 selected ‘prefer not to say’). As in our previous experiments, participants were reimbursed with a base payment of £1.50 (based on an estimate of £9.00/hr), plus an additional bonus payment of £0.01 per 3 points scored (mean total reward £1.92, min £1.80, max £2.04). Mean experiment duration was 9 m 23 s (*SD* 3 m 57 s).

#### Design.

**Figure 7.**
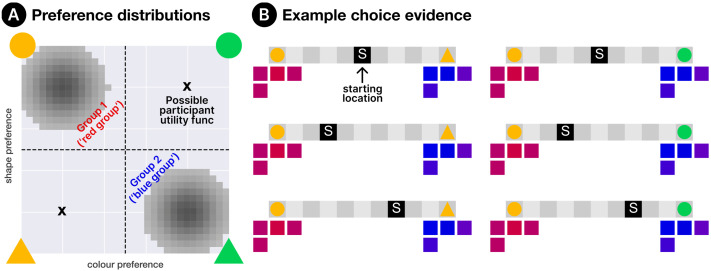
An illustration of the group preference distributions for Experiment 3, along with some example choice evidence (for 4 agents per group). The two possible participant utility functions are also shown. The *ϕ* (explicit cue) distributions are the same as in Experiment 2.

We manipulated just one factor between participants. Similar to factor 2 in the previous experiment, participants’ explicit cue was either ‘invisible’ (grey), or ‘arbitrary’ (set to the mean of the group whose *shape* preference the participant shared). As before, the agent population is organised into two groups. Figure 7 illustrates the group distributions over utility functions and explicit cue values. Membership of Group 1 (the ‘red’ group) is associated with a preference for *both* circles and yellow items; of Group 2 (the ‘blue’ group) with the opposite preferences, for triangles and green items. Crucially, participants’ utility functions are set to be in one of the two *other* quadrants–triangles and yellow, or circles and green. Utility function weights are additive: an agent with preferences in the ‘yellow + circles’ quadrant will receive high reward for a yellow circle, medium reward for a yellow triangle or green circle, and low reward for a green triangle. The group distributions for explicit cue (*ϕ*) were the same as in Experiment 2, and as before, participants were given no direct instruction as to the existence of any social groups within the environment.

#### Procedure.

The procedure for Experiment 3 was very similar to that for Experiment 2, involving controlling an avatar through a gridworld environment to complete a series of simple choice tasks. Participants first played through an exploration phase where they learned about their own (assigned) utility functions by collecting items with no other agents present. They then played through a phase based on the ’Unknown agents’ phase from Experiment 2. In this phase, they first observed one set of agents making choices between visible items (designed to allow inference of the group distributions). They were then faced with a final sequence involving items hidden in ‘mystery boxes’, consisting of four repeats each of two levels–one level with items that differed only on colour, and one with items that differed only on shape. Importantly, participants were told before each level which dimension the two hidden items differed on. As in Experiment 2, the demonstrator agents present in these final levels were new/unfamiliar–meaning participants had no direct (behavioural) evidence of their preferences, and instead had to rely on the visual cues *ϕ* and what they had learned about the two social groups. Also as in Experiment 2, participants and simulated agents lost one point for each step taken in the gridworld.

### Results

Participants’ trajectories were recorded and analysed following the same procedure as in the two previous experiments (see Experiment 1: Preference Inference and Selective Social Learning section). As before, we compare participants’ relative selectivity to each of the two groups against both a naÏve cue-based ingroup strategy, and our rational model. For this experiment, we extend our rational model slightly to incorporate an additional context-dependent feature weighting in the calculation of similarity between agents’ utility functions:

$${\text{sim}}_{\text{contextual}}\left(U^{\text{ego}},U^{\left(m\right)}|c\right)=\sum_{i}c_{i}|U_{i}^{\text{ego}}-0.5|\left(1-|U_{i}^{\text{ego}}-U_{i}^{\left(m\right)}|\right)$$ (16)

where **c** is a binary vector denoting whether each feature dimension *i* appears in the current choice context. Other than this tweak to the similarity function, the rational model is exactly the same as described in Rational Group-Based Selection section. For this experiment we also compare to an additional model that implements the ‘agreement frequency’ strategy (S5 in Table 1). In this model, the social learner does use the evidence presented of group members’ item choices to select a social learning target—but rather than inferring the preference distributions and then applying them selectively in context, they weight each group by how often they’ve agreed with that group’s members over the history of visible choices, and then use those weights to select social learning targets regardless of context. To ensure that this model makes distinct predictions to both the naÏve and full rational models, we set the ‘evidence’ levels up to contain 3 choices between different-coloured items and only 2 choices between different-shaped items. A participant following this strategy in the ‘test’ levels should therefore be consistently biased towards learning from agents in the group which shares their colour preference^8^.

**Figure 8.**
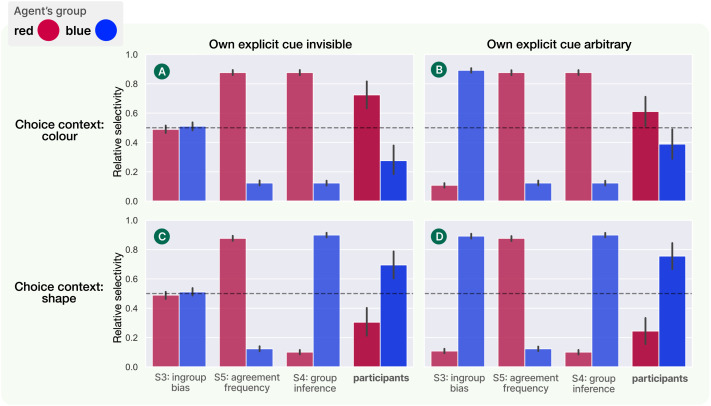
Results of Experiment 3. As before, bar heights measure relative selectivity in favour of each of two available demonstrators (one from each social group), and error bars give 95% confidence intervals. Participant behaviour is compared against the ingroup bias model (S3 in Table 1), our rational group inference model (S4) and the ‘agreement frequency’ model (S5). The rational model is the only one to capture the pattern of participants’ choices across all conditions.

Figure 8 shows the output of our analysis and model comparison for Experiment 3. As before, we collapse the counterbalancing and present the results as though all participants are aligned with the ‘red’ group on colour preference and the ‘blue’ group on shape preference. We can see that across both the ‘own cue invisible’ and ‘own cue arbitrary’ conditions, participants were sensitive to the two different choice contexts, correctly favouring the red-group social learning target in a choice based on item colour (A: relative selectivity 0.724 (63/87 trajectories), *p* < 0.001; B: 0.611 (55/90), *p* = 0.0263) and the blue-group social learning target in a choice based on item shape (C: 0.690 (60/87), *p* < 0.001; D: 0.756 (68/90), *p* < 0.001). While this trend held across both conditions, we note that it was weaker for the ‘own cue arbitrary’ condition, where participants overall showed more tendency towards imitating the agent from the group matching their own explicit cue (and with whom they shared a shape preference). Comparing to the three models, we see that our rational group-based model (S4 in Table 1) is the only one to predict the trend of participants’ behaviour across all combinations of condition and choice context. Most importantly, the ‘agreement frequency’ model (S5), which was not excluded by the results of Experiment 2, failed to account for the data from the shape context.

**Figure 9.**
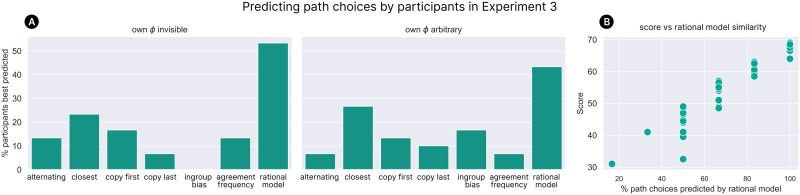
(A) The percentage of participants in Experiment 3 best fit by each of various strategies (where a participant is considered to be best fit by a strategy if that strategy correctly predicts the highest proportion of their path choices). (B) The relationship between how well each participant’s behaviour was predicted by our rational model, and the score they achieved on the test levels of the task.

As in Experiment 2, we also compared our rational model to a set of simpler heuristic strategies by predicting participants’ individual path choices. As Figure 9A shows, a plurality of participants in Experiment 3 were fit best by our rational model. We also found, similar to Experiment 2, that participants whose behaviour was better predicted by our rational model achieved higher task scores (Figure 9B).

Taken together, the results of Experiments 2 and 3 demonstrate that people make rational use of cues to group identity to guide their selective social learning, rather than relying on simpler heuristics.

## GENERAL DISCUSSION

The potential benefits of social learning have long been appreciated by researchers across multiple fields. By learning from others, individuals can reduce the risk, effort and opportunity cost associated with exploration, enabling safer and more efficient adaptation to their environment, or to a new task domain. Similarly, it is well established that agents should in general be selective rather than indiscriminate in whom they choose to learn from, since not everyone in their environment will be equally suitable as a demonstrator of adaptive behaviour.

However, much of the prior literature on social learning strategies deals principally with demonstrator attributes such as competence, success or social status. Less well-understood is the role played by preferences, as well as by learner and demonstrator group identity. Prior work suggests that people follow an ingroup bias in choosing whom to learn from (Buttelmann et al., 2013; Golkar et al., 2015; Howard et al., 2015; Pető et al., 2018), even in settings where group identity is not meaningful (Montrey & Shultz, 2022). At the same time, other instances of selectivity can be viewed as people favouring learning from outgroup over ingroup individuals, such as children favouring information from teachers rather than from their peers (Rakoczy et al., 2010; Seehagen & Herbert, 2011).

In this paper, we have examined the role of group identity in selective social learning under preference variation. Specifically, we sought to understand whether people can go beyond a simple ingroup bias and make flexible use of group identity cues to identify the most rewarding demonstrator in different contexts. While some prior evidence exists that children account for preference similarity in demonstrator selection (Fawcett & Markson, 2010), our work provides the first *formal* account of this behaviour—first via rational inference of preferences at the individual level, and then by making use of social structure and group identity to generalise to newly encountered agents.

Our primary contribution to the literature is in describing and unpacking this problem at the computational level. Using a variation on the framework of Markov Decision Processes, we first outlined the *optimal* approach to selective social learning under preference variation. Taking inspiration from prior work on modelling how people infer others’ preferences (Baker et al., 2009, 2017; Jern et al., 2017; Lucas et al., 2009, 2014), we then set up a series of experiments that allowed us to compare people’s social learning behaviour with our rational account. While the results of these experiments do shed some light on how people use behavioural and featural evidence to select social learning targets under uncertainty, the findings should not be particularly controversial, and should be viewed primarily as a demonstration and validation of the formal account which is our main contribution. These experiments also allowed us to refine our account in the rational analysis tradition (Anderson, 1990), by considering how human social learners might enact computationally feasible approximations to the optimal approach.

Our empirical contribution focuses mainly on the question of how people use explicit cues to group identity in selective social learning under preference variation, comparing a model of naïve ingroup bias against a novel rational model based on inferring the underlying relationship between group identity and preferences. Across three behavioural experiments in a choice-under-uncertainty setting, we showed that our rational model predicted people’s social learning behaviour better than simpler alternatives. Our model outperformed these other accounts in virtue of its ability to capture how participants used (or ignored) group cues depending on whether they were useful, uninformative or actively misleading within a given choice environment. While individual experiments may leave room for alternative accounts, we believe that when taken as a whole, our pattern of results provides convincing evidence that people follow a rational approach to selecting social learning targets under preference variation. In particular, our results demonstrate that people are sensitive to the contextual significance of group identity markers when considering the suitability of different potential demonstrators, rather than relying only on a simple ingroup bias. Although limited by the arbitrary nature of the ingroup-outgroup distinction we employ, our group-level account may help to reconcile a seemingly incongruent picture of ingroup vs outgroup selectivity in human social learning, in which people seem to favour learning from ingroup members in some cases (Buttelmann et al., 2013; Frazier et al., 2012; Kinzler et al., 2011; Ma & Woolley, 2013; Shutts et al., 2010; Taylor, 2013) and outgroup members in others (Jaswal & Neely, 2006; Rakoczy et al., 2010; Seehagen & Herbert, 2011; Wood et al., 2012). These results can all be explained by a general social learning strategy that uses group identity in a way dependent on its contextual significance, which is a key component of our rational model.

We believe that this paper makes a valuable contribution to understanding a relatively under-explored angle of selective social learning. However, there are limitations that we hope to address in future work. Although in our initial theoretical discussion (Optimal Selective Social Learning section) we considered the joint objective of finding social learning targets that maximise both preference similarity and competence (i.e., minimise decision noise), our experimental validations dealt only with the former. An obvious extension therefore is to consider agent populations that vary in both values and competence, and so test whether people can account for both factors simultaneously in their selection of social learning targets^9^. In particular, it seems that prestige-biased social learning might fit well within a similar rational framework, where learners must infer over time the relationship between observable prestige markers and underlying ability in different domains—just as they do in our model between group labels and item preferences. Future work might also consider how social learners balance different selection criteria within a broader rational strategy (e.g., how to weight preference similarity vs competence when no single demonstrator is ‘ideal’ along both dimensions).

Perhaps the main ecological limitation of our experiments was that we considered only a simple social structure, with just two fixed non-overlapping groups. This allowed us to keep our translation from formalism to behavioural predictions explicit and exhaustive. However, real-world social structure is rarely this straightforward, and obvious extensions would be to consider increasing the number of groups, introducing hierarchical subgroups and non-exclusivity, and allowing group structure to evolve over time. Relatedly, the groups in our experimental setting were entirely artificial and context-specific—participants had no prior reason to consider themselves aligned with or against the ‘red’ or ‘blue’ agent groups. While this was advantageous in minimising the effects of any pre-existing stereotypes or biases on participants’ behaviour, it does raise the question of how our results would generalise to a setting with more familiar or persistent dimensions of group identity. One approach might be to extend our model to consider people as assigning demonstrator weights *w*^(*m*)^ that are a combination of both an ingroup bias weight and a rational-inference-informed weight, balanced in such a way that the influence of the ingroup bias is a function of both how entrenched the groups are, as well as the amount and quality of context-relevant behavioural evidence the learner has access to. In our experiments, where groups are ‘shallow’ and choice evidence is unambiguous, this model would effectively reduce to what we have now; in more realistic settings, it would allow for ingroup biases to play a bigger role. While directly testing an account like this would be challenging, follow-up work that examines how people’s usage of group identity in selective social learning changes depending on how well-established or culturally significant those groups are deemed to be could certainly prove interesting. On this point, we note recent work by Montrey and Shultz (2022), which also studied social learning in a minimal groups paradigm (where, as in our experiments, participants were randomly assigned to colour-coded groups) using a task with no difference in reward structure between groups. The authors found that a robust ingroup bias emerged despite participants having no prior reason to consider either group as a more reliable source of demonstrators—suggesting that the relative failure of the naïve ingroup bias model to account for our results does not result simply from our use of arbitrary groups.

A final limitation of the current work is that we restrict ourselves to a fundamentally individualistic setting—while our environments are ‘social’ in the sense of featuring multiple agents and social groups, those agents never share the environment at the same time, and so each individual’s actions can affect only themselves. In the real world this is rarely the case; we must balance satisfying our own preferences while abiding by moral/social norms and avoiding conflict with others. Future work might look at how competitive and cooperative dynamics influence the role of group identity in people’s selective social learning behaviour.

While not a limitation as such, it is worth emphasising that our account sits purely at the computational level, and does not shed any particular light on the algorithmic processes that might underlie the rational strategy. A process model of group-based selective social learning would face additional complexities, such as how people distinguish representations of their own vs others’ preferences (Ereira et al., 2018), and how people’s own preferences influence or warp their interpretations of others’ choice behaviour (Tarantola et al., 2017). More fundamentally, it would have to account for how people deal with the complexity of the inference task in environments with higher preference dimensionality, large agent numbers, or significant behavioural noise.

We conclude by touching briefly on potential broader implications for future work. One possible interpretation of our analysis is as offering a rational account *for* ingroup bias in social learning: while the conditions in our experimental setting were designed specifically to produce disagreement between a naïve ingroup bias and a rational inference-based model, this contradiction is likely not representative of real-world settings, where we expect that the two strategies often make similar selections. Considering the potential costs of the full inferential approach, the naïve ingroup bias may be somestimes be viewed as a ‘usually good enough’ approximation to the optimal strategy (Simon, 1956). Under this view, we might expect people to rely on an ingroup bias strategy unless they find themselves in a setting where it is producing poor outcomes or they receive some other evidence that such an approach is insufficient or misguided. This sort of resource-rational strategy-switching is not something that our current experimental setup is able to say much about; but we think it is an interesting premise for subsequent study.

Social learning is of course just one context in which people use group-level representations of others to guide their decision-making. We suggest that the approach taken here might be fruitfully applied to accounting for group-based biases in other settings, such as in moral or political decision-making. It may be the case that many group-based biases are in fact low-cost approximations to rational strategies for problems of social cognition under limited information.

## Notes

^1^ Although we note that while we model agents as being Boltzmann-rational, they needn’t actually implement such a policy in practice; and could instead be rational but with unobservable contributors to their utility functions.^2^ For example, whether or not you share someone’s favourite genre of cinema does not determine whether you’ll find their choice of restaurant rewarding.^3^ Since the spatial dynamics are not strictly necessary to our design, we could have alternatively used a static bandit-like interface—but we argue that the game-like setup is more engaging for participants.^4^ We used the CIELAB colourspace for perceptual uniformity, with each agent’s colour given as (25, 95, −*x* * 128) for some *x* ∈ [0, 1]. This produces a uniform spectrum from red at *x* = 0 to blue at *x* = 1.^5^ For example, if a participant cares only about item colour, and the two explicit groups correspond respectively to a preference for circles and triangles, then it does not make sense to say that the participant’s explicit cue is matched or mismatched to their utility function; if present, it can only be arbitrary. Likewise, if the explicit groups *do* align with the dimension that the participant cares about, then their explicit cue can only be matched or mismatched, and not arbitrary.^6^ The specific matching of agent names to groups was randomised, as was the order in which the agents were observed in the environment. This was done to prevent participants from being able to rely on any alphabetical or presentation-order heuristics.^7^ Note that a given participant may be best-captured by more than one strategy (e.g., if two strategies both predict 75% of their choices correctly) and so bar heights don’t necessarily sum to 100.^8^ In principle, we could have a version of the agreement frequency model which does incorporate choice context, making judgements of the form “I’ve agreed more with group X on shape choices” or “I’ve agreed more with group Y on colour choices”—but since this essentially just makes implicit the same group-preference association at the core of our rational model, we don’t try to test it here.^9^ We note that lots of prior work has studied competence and related factors (e.g., knowledge, reliability) in social learning settings already (Brody & Stoneman, 1985; Jaswal & Malone, 2007; Jaswal & Neely, 2006; Koenig & Harris, 2005).

## APPENDIX A: GLOSSARY OF SYMBOLS

**Table A1.** Glossary of mathematical symbols used in the present work

Symbol	Definition
𝒮	State space of Markov Decision Process (MDP)
𝒜	Action space of MDP
*T* : 𝒮 × 𝒜 × 𝒮 → [0, 1]	Transition function of MDP
*r* : 𝒮 × 𝒜 → ℝ	Global reward function of MDP
*π* : 𝒮 × 𝒜 → [0, 1]	Policy of agent acting in MDP
*V*_*π*_(·) : 𝒮 → ℝ	State-value function under policy *π*
*Q*_*π*_(·, ·) : 𝒮 × 𝒜 → ℝ	Action-value function under policy *π*
*γ* ∈ [0, 1]	Discount factor for MDP
*τ*^(*m*)^ = {(*s*, *a*)}	Trajectory executed in MDP by agent *m*
*β*^(*m*)^ ∈ ℝ^+^	Decision noise of agent *m* acting under Boltzmann-rational policy
*U*^(*m*)^ : 𝒮 × 𝒜 → ℝ	Agent-specific utility function (of agent *m*)
sim(*U*^(*m*)^, *U*^(*n*)^) ∈ ℝ	Similarity between utility functions of agents *m* and *n*
*w*^(*m*)^ ∈ ℝ	Weight assigned to agent *m* (as potential demonstrator) by selective social learner
*O*^(*m*)^ = {*τ*}	Set of observed behaviour (trajectories) from agent *m*
*F*(*τ*_*i*_, *τ*_*j*_) ∈ ℝ^+^	Discrete Fréchet distance between trajectories *τ*_*i*_, *τ*_*j*_
*s*(*τ*_*i*_, *τ*_*j*_) ∈ ℝ^+^	Similarity between trajectories *τ*_*i*_, *τ*_*j*_
*z*^(*m*)^ ∈ ℤ^+^	Latent social group assignment of agent *m*
*ϕ* ^(*m*)^	Explicit observable cue expressed by agent *m* (noisy signal of *z*^(*m*)^)
*θ* ^*k*^	Latent parameters associated with social group *k* (parameterises distributions over both *U* and *ϕ* for group members)
*ρ*	Group mixture weights in Dirichlet process mixture model

## References

[bib1] Abramovitch, R., Corter, C. M., & Pepler, D. J. (1980). Observations of mixed-sex sibling dyads. Child Development, 51(4), 1268–1271. 10.1111/j.1467-8624.1980.tb02679.x

[bib2] Alanqary, A., Lin, G. Z., Le, J., Zhi-Xuan, T., Mansinghka, V. K., & Tenenbaum, J. B. (2021). Modeling the mistakes of boundedly rational agents within a Bayesian theory of mind. arXiv. 10.48550/arXiv.2106.13249

[bib3] Anderson, J. R. (1990). The adaptive character of thought. Psychology Press. 10.4324/9780203771730

[bib4] Arulkumaran, K., Deisenroth, M. P., Brundage, M., & Bharath, A. A. (2017). Deep reinforcement learning: A brief survey. IEEE Signal Processing Magazine, 34, 26–38. 10.1109/MSP.2017.2743240

[bib6] Baker, C. L., Jara-Ettinger, J., Saxe, R., & Tenenbaum, J. B. (2017). Rational quantitative attribution of beliefs, desires and percepts in human mentalizing. Nature Human Behaviour, 1(4), 0064. 10.1038/s41562-017-0064

[bib7] Baker, C. L., Saxe, R., & Tenenbaum, J. B. (2009). Action understanding as inverse planning. Cognition, 113(3), 329–349. 10.1016/j.cognition.2009.07.005, 19729154

[bib8] Bamford, C. D., Jiang, M., Samvelyan, M., & Rocktäschel, T. (2022). GriddlyJS: A web IDE for reinforcement learning. arXiv. 10.48550/arXiv.2207.06105

[bib9] Baron, A. S., & Dunham, Y. (2015). Representing ‘us’ and ‘them’: Building blocks of intergroup cognition. Journal of Cognition and Development, 16, 780–801. 10.1080/15248372.2014.1000459

[bib10] Bhui, R., Lai, L., & Gershman, S. J. (2021). Resource-rational decision making. Current Opinion in Behavioral Sciences, 41, 15–21. 10.1016/j.cobeha.2021.02.015

[bib11] Birch, S. A. J., Vauthier, S. A., & Bloom, P. (2008). Three- and four-year-olds spontaneously use others’ past performance to guide their learning. Cognition, 107(3), 1018–1034. 10.1016/j.cognition.2007.12.008, 18295193

[bib12] Bobu, A., Scobee, D. R. R., Fisac, J. F., Sastry, S. S., & Dragan, A. D. (2020). LESS is more: Rethinking probabilistic models of human behavior. In Proceedings of the 2020 ACM/IEEE International Conference on Human-Robot Interaction (pp. 429–437). Association for Computing Machinery. 10.1145/3319502.3374811

[bib13] Boyd, R., & Richerson, P. J. (1985). Culture and the evolutionary process. University of Chicago Press.

[bib14] Brody, G. H., & Stoneman, Z. (1985). Peer imitation: An examination of status and competence hypotheses. Journal of Genetic Psychology, 146, 161–170. 10.1080/00221325.1985.9914443

[bib15] Buttelmann, D., Zmyj, N., Daum, M. M., & Carpenter, M. (2013). Selective imitation of in-group over out-group members in 14-month-old infants. Child Development, 84(2), 422–428. 10.1111/j.1467-8624.2012.01860.x, 23006251

[bib16] Chudek, M., Heller, S., Birch, S., & Henrich, J. (2012). Prestige-biased cultural learning: Bystander’s differential attention to potential models influences children’s learning. Evolution and Human Behavior, 33, 46–56. 10.1016/j.evolhumbehav.2011.05.005

[bib17] Coussi-Korbel, S., & Fragaszy, D. M. (1995). On the relation between social dynamics and social learning. Animal Behaviour, 50, 1441–1453. 10.1016/0003-3472(95)80001-8

[bib18] Cunningham, S. J., Hutchison, J., Ellis, N., Hezelyova, I., & Wood, L. A. (2023). The cost of social influence: Own-gender and gender-stereotype social learning biases in adolescents and adults. PLOS ONE, 18, e0290122. 10.1371/journal.pone.0290122, 37566606 PMC10420340

[bib19] Dennett, D. C. (1987). The intentional stance. MIT Press.

[bib20] Devine, P. G. (1989). Stereotypes and prejudice: Their automatic and controlled components. Journal of Personality and Social Psychology, 56, 5–18. 10.1037/0022-3514.56.1.5

[bib21] Diesendruck, G., & HaLevi, H. (2006). The role of language, appearance, and culture in children’s social category-based induction. Child Development, 77(3), 539–553. 10.1111/j.1467-8624.2006.00889.x, 16686787

[bib22] Duffy, G. A., Pike, T. W., & Laland, K. N. (2009). Size-dependent directed social learning in nine-spined sticklebacks. Animal Behaviour, 78, 371–375. 10.1016/j.anbehav.2009.05.015

[bib23] Dunham, Y. (2018). Mere membership. Trends in Cognitive Sciences, 22, 780–793. 10.1016/j.tics.2018.06.004, 30119749

[bib24] Eiter, T., & Mannila, H. (1994). Computing discrete Fréchet distance (Technical report CD-TR 94/64). Information Systems Department, Technical University of Vienna.

[bib25] Ereira, S., Dolan, R. J., & Kurth-Nelson, Z. (2018). Agent-specific learning signals for self–other distinction during mentalising. PLOS Biology, 16(4), e2004752. 10.1371/journal.pbio.2004752, 29689053 PMC5915684

[bib26] Evans, O., Stuhlmüller, A., & Goodman, N. D. (2016). Learning the preferences of ignorant, inconsistent agents. In Proceedings of the 30th AAAI Conference on Artificial Intelligence (pp. 323–329). AAAI Press. 10.1609/aaai.v30i1.10010

[bib27] Fawcett, C. A., & Markson, L. (2010). Children reason about shared preferences. Developmental Psychology, 46(2), 299–309. 10.1037/a0018539, 20210491

[bib28] Finn, C., Levine, S., & Abbeel, P. (2016). Guided cost learning: Deep inverse optimal control via policy optimization. In Proceedings of the 33rd International Conference on Machine Learning (Vol. 48, pp. 49–58). JMLR.org.

[bib29] Fiske, S. T., & Neuberg, S. L. (1989). Category-based and individuating processes as a function of information and motivation: Evidence from our laboratory. In D. Bar-Tal, C. F. Graumann, A. W. Kruglanski, & W. Stroebe (Eds.), Stereotyping and prejudice: Changing conceptions (pp. 83–103). Springer. 10.1007/978-1-4612-3582-8_4

[bib30] Fiske, S. T., & Neuberg, S. L. (1990). A continuum of impression formation, from category-based to individuating processes: Influences of information and motivation on attention and interpretation. Advances in Experimental Social Psychology, 23, 1–74. 10.1016/S0065-2601(08)60317-2

[bib31] Foster-Hanson, E., Roberts, S. O., Gelman, S. A., & Rhodes, M. (2021). Categories convey prescriptive information across domains and development. Journal of Experimental Child Psychology, 212, 105231. 10.1016/j.jecp.2021.105231, 34358722 PMC8666967

[bib32] François-Lavet, V., Henderson, P., Islam, R., Bellemare, M. G., & Pineau, J. (2018). An introduction to deep reinforcement learning. Foundations and Trends® in Machine Learning, 11(3–4), 219–354. 10.1561/2200000071

[bib33] Frazier, B. N., Gelman, S. A., Kaciroti, N. A., Russell, J. W., & Lumeng, J. C. (2012). I’ll have what she’s having: The impact of model characteristics on children’s food choices. Developmental Science, 15(1), 87–98. 10.1111/j.1467-7687.2011.01106.x, 22251295 PMC3261590

[bib34] Gelpí, R. A., & Buchsbaum, D. (2023). Children as cultural explorers: How imitation, pedagogy, and selective trust prepare children for learning in the cultural niche. PsyArXiv. 10.31234/osf.io/f4cwu

[bib35] Gergely, G., Nádasdy, Z., Csibra, G., & Bíró, S. (1995). Taking the intentional stance at 12 months of age. Cognition, 56, 165–193. 10.1016/0010-0277(95)00661-H, 7554793

[bib36] Gershman, S. J., & Blei, D. M. (2012). A tutorial on Bayesian nonparametric models. Journal of Mathematical Psychology, 56, 1–12. 10.1016/j.jmp.2011.08.004

[bib37] Gershman, S. J., Pouncy, H. T., & Gweon, H. (2017). Learning the structure of social influence. Cognitive Science, 41(Suppl. 3), 545–575. 10.1111/cogs.12480, 28294384

[bib38] Golkar, A., Castro, V., & Olsson, A. (2015). Social learning of fear and safety is determined by the demonstrator’s racial group. Biology Letters, 11(1), 20140817. 10.1098/rsbl.2014.0817, 25631229 PMC4321149

[bib5] Goodman, N. D., Baker, C. L., & Tenenbaum, J. B. (2009). Cause and intent: Social reasoning in causal learning In N. Taatgen & H. van Rijn (Eds.), Proceedings of the 31st Annual Conference of the Cognitive Science Society (pp. 2759–2764). Cognitive Science Society.

[bib39] Gopnik, A., & Meltzoff, A. N. (1997). Words, thoughts, and theories. MIT Press. 10.7551/mitpress/7289.001.0001

[bib40] Gramzow, R. H., Gaertner, L., & Sedikides, C. (2001). Memory for in-group and out-group information in a minimal group context: The self as an informational base. Journal of Personality and Social Psychology, 80(2), 188–205. 10.1037/0022-3514.80.2.188, 11220440

[bib41] Greenwald, A. G., Pickrell, J. E., & Farnham, S. D. (2002). Implicit partisanship: Taking sides for no reason. Journal of Personality and Social Psychology, 83(2), 367–379. 10.1037/0022-3514.83.2.367, 12150234

[bib42] Griffiths, T. L., Canini, K. R., Sanborn, A. N., & Navarro, D. J. (2007). Unifying rational models of categorization via the hierarchical Dirichlet process. In D. S. McNamara & J. G. Trafton (Eds.), Proceedings of the 29th Annual Conference of the Cognitive Science Society (pp. 323–328). Erlbaum.

[bib43] Heider, F., & Simmel, M. L. (1944). An experimental study of apparent behavior. American Journal of Psychology, 57, 243–259. 10.2307/1416950

[bib44] Henrich, J. (2018). The secret of our success: How culture is driving human evolution, domesticating our species, and making us smarter. Princeton University Press. 10.1515/9781400873296

[bib45] Henrich, J., & Gil-White, F. J. (2001). The evolution of prestige: Freely conferred deference as a mechanism for enhancing the benefits of cultural transmission. Evolution and Human Behavior, 22(3), 165–196. 10.1016/s1090-5138(00)00071-4, 11384884

[bib46] Ho, J., & Ermon, S. (2016). Generative adversarial imitation learning. In Proceedings of the 30th International Conference on Neural Information Processing Systems (pp. 4572–4580). Curran Associates Inc.

[bib47] Horner, V., Proctor, D., Bonnie, K. E., Whiten, A., & de Waal, F. B. M. (2010). Prestige affects cultural learning in chimpanzees. PLoS ONE, 5(5), e10625. 10.1371/journal.pone.0010625, 20502702 PMC2873264

[bib48] Howard, L. H., Henderson, A. M. E., Carrazza, C., & Woodward, A. L. (2015). Infants’ and young children’s imitation of linguistic in-group and out-group informants. Child Development, 86(1), 259–275. 10.1111/cdev.12299, 25263528 PMC4358791

[bib49] Icard, T. F. (2023). Resource rationality (Unpublished manuscript).

[bib50] Jara-Ettinger, J. (2019). Theory of mind as inverse reinforcement learning. Current Opinion in Behavioral Sciences, 29, 105–110. 10.1016/j.cobeha.2019.04.010

[bib51] Jara-Ettinger, J., Gweon, H., Schulz, L. E., & Tenenbaum, J. B. (2016). The naïve utility calculus: Computational principles underlying commonsense psychology. Trends in Cognitive Sciences, 20(8), 589–604. 10.1016/j.tics.2016.05.011, 27388875

[bib52] Jaswal, V. K., & Malone, L. S. (2007). Turning believers into skeptics: 3-year-olds’ sensitivity to cues to speaker credibility. Journal of Cognition and Development, 8, 263–283. 10.1080/15248370701446392

[bib53] Jaswal, V. K., & Neely, L. A. (2006). Adults don’t always know best: Preschoolers use past reliability over age when learning new words. Psychological Science, 17, 757–758. 10.1111/j.1467-9280.2006.01778.x, 16984291

[bib54] Jern, A., Lucas, C. G., & Kemp, C. (2017). People learn other people’s preferences through inverse decision-making. Cognition, 168, 46–64. 10.1016/j.cognition.2017.06.017, 28662485 PMC5572562

[bib55] Kaaronen, R. O. (2020). Mycological rationality: Heuristics, perception and decision-making in mushroom foraging. Judgment and Decision Making, 15(5), 630–647. 10.1017/S1930297500007841

[bib56] Kaaronen, R. O., Manninen, M. A., & Eronen, J. T. (2023). Rules of thumb, from Holocene to Anthropocene. Anthropocene Review, 10(3), 685–709. 10.1177/20530196221149105

[bib57] Kalish, C. W., & Lawson, C. A. (2008). Development of social category representations: Early appreciation of roles and deontic relations. Child Development, 79(3), 577–593. 10.1111/j.1467-8624.2008.01144.x, 18489414

[bib58] Kinzler, K. D., Corriveau, K. H., & Harris, P. L. (2011). Children’s selective trust in native-accented speakers. Developmental Science, 14(1), 106–111. 10.1111/j.1467-7687.2010.00965.x, 21159092

[bib59] Kinzler, K. D., Dupoux, E., & Spelke, E. S. (2007). The native language of social cognition. Proceedings of the National Academy of Sciences, 104, 12577–12580. 10.1073/pnas.0705345104, 17640881 PMC1941511

[bib60] Kober, J., Bagnell, J. A., & Peters, J. (2013). Reinforcement learning in robotics: A survey. International Journal of Robotics Research, 32, 1238–1274. 10.1177/0278364913495721

[bib61] Koenig, M., & Harris, P. L. (2005). Preschoolers mistrust ignorant and inaccurate speakers. Child Development, 76(6), 1261–1277. 10.1111/j.1467-8624.2005.00849.x, 16274439

[bib62] Kushnir, T., Xu, F., & Wellman, H. M. (2010). Young children use statistical sampling to infer the preferences of other people. Psychological Science, 21, 1134–1140. 10.1177/0956797610376652, 20622142 PMC3785083

[bib63] Lai, L., & Gershman, S. J. (2024). Human decision making balances reward maximization and policy compression. PLOS Computational Biology, 20(4), e1012057. 10.1371/journal.pcbi.1012057, 38669280 PMC11078408

[bib64] Laland, K. N. (2004). Social learning strategies. Learning & Behavior, 32, 4–14. 10.3758/BF03196002, 15161136

[bib65] Lau, T., Gershman, S. J., & Cikara, M. (2020). Social structure learning in human anterior insula. eLife, 9, e53162. 10.7554/eLife.53162, 32067635 PMC7136019

[bib66] Lev-Ari, S., & Keysar, B. (2010). Why don’t we believe non-native speakers? The influence of accent on credibility. Journal of Experimental Social Psychology, 46, 1093–1096. 10.1016/j.jesp.2010.05.025

[bib67] Liberman, Z., Woodward, A. L., & Kinzler, K. D. (2017). The origins of social categorization. Trends in Cognitive Sciences, 21, 556–568. 10.1016/j.tics.2017.04.004, 28499741 PMC5605918

[bib68] Liberman, Z., Woodward, A. L., Sullivan, K. R., & Kinzler, K. D. (2016). Early emerging system for reasoning about the social nature of food. Proceedings of the National Academy of Sciences, 113, 9480–9485. 10.1073/pnas.1605456113, 27503878 PMC5003271

[bib69] Lieder, F., & Griffiths, T. L. (2019). Resource-rational analysis: Understanding human cognition as the optimal use of limited computational resources. Behavioral and Brain Sciences, 43, e1. 10.1017/S0140525X1900061X, 30714890

[bib70] Lucas, C. G., Griffiths, T. L., Xu, F., & Fawcett, C. (2009). A rational model of preference learning and choice prediction by children. In D. Koller, D. Schuurmans, Y. Bengio, & L. Bottou (Eds.), Advances in neural information processing systems 21 (pp. 985–992).

[bib71] Lucas, C. G., Griffiths, T. L., Xu, F., Fawcett, C., Gopnik, A., Kushnir, T., Markson, L., & Hu, J. C. (2014). The child as econometrician: A rational model of preference understanding in children. PLoS ONE, 9(3), e92160. 10.1371/journal.pone.0092160, 24667309 PMC3965422

[bib72] Ma, L., & Woolley, J. D. (2013). Young children’s sensitivity to speaker gender when learning from others. Journal of Cognition and Development, 14, 100–119. 10.1080/15248372.2011.638687

[bib73] Macrae, C. N., & Bodenhausen, G. V. (2000). Social cognition: Thinking categorically about others. Annual Review of Psychology, 51, 93–120. 10.1146/annurev.psych.51.1.93, 10751966

[bib74] Markman, E. M. (1989). Categorization and naming in children: Problems of induction. MIT Press.

[bib75] Meltzoff, A. N. (2007). ‘Like me’: A foundation for social cognition. Developmental Science, 10(1), 126–134. 10.1111/j.1467-7687.2007.00574.x, 17181710 PMC1852489

[bib76] Mesoudi, A., & O’Brien, M. J. (2008). The cultural transmission of Great Basin projectile-point technology II: An agent-based computer simulation. American Antiquity, 73, 627–644. 10.1017/S0002731600047338

[bib77] Montrey, M., & Shultz, T. R. (2022). Copy the in-group: Group membership trumps perceived reliability, warmth, and competence in a social-learning task. Psychological Science, 33(1), 165–174. 10.1177/09567976211032224, 34939477 PMC13038129

[bib78] Nisbett, R. E., Krantz, D. H., Jepson, C., & Kunda, Z. (1983). The use of statistical heuristics in everyday inductive reasoning. Psychological Review, 90, 339–363. 10.1037/0033-295X.90.4.339

[bib79] Osherson, D. N., Smith, E. E., Wilkie, O., López, A., & Shafir, E. (1990). Category-based induction. Psychological Review, 97, 185–200. 10.1037/0033-295X.97.2.185

[bib80] Pető, R., Elekes, F., Oláh, K., & Király, I. (2018). Learning how to use a tool: Mutually exclusive tool–function mappings are selectively acquired from linguistic in-group models. Journal of Experimental Child Psychology, 171, 99–112. 10.1016/j.jecp.2018.02.007, 29567562

[bib81] Puterman, M. L. (1994). Markov decision processes: Discrete stochastic dynamic programming. John Wiley & Sons, Inc. 10.1002/9780470316887

[bib82] Rakoczy, H., Hamann, K., Warneken, F., & Tomasello, M. (2010). Bigger knows better: Young children selectively learn rule games from adults rather than from peers. British Journal of Developmental Psychology, 28(4), 785–798. 10.1348/026151009x479178, 21121467

[bib83] Ramachandran, D., & Amir, E. (2007). Bayesian inverse reinforcement learning. In Proceedings of the 20th International Joint Conference on Artificial Intelligence (pp. 2586–2591). Morgan Kaufmann Publishers Inc.

[bib84] Rawls, J. (1971). A theory of justice. The Belknap Press of Harvard University Press.

[bib85] Rendell, L., Fogarty, L., & Laland, K. N. (2010). Rogers’ paradox recast and resolved: Population structure and the evolution of social learning strategies. Evolution, 64(2), 534–548. 10.1111/j.1558-5646.2009.00817.x, 19674093

[bib86] Repacholi, B. M., & Gopnik, A. (1997). Early reasoning about desires: Evidence from 14- and 18-month-olds. Developmental Psychology, 33(1), 12–21. 10.1037/0012-1649.33.1.12, 9050386

[bib87] Rogers, A. R. (1988). Does biology constrain culture? American Anthropologist, 90, 819–831. 10.1525/aa.1988.90.4.02a00030

[bib88] Sanborn, A. N., Griffiths, T. L., & Navarro, D. J. (2010). Rational approximations to rational models: Alternative algorithms for category learning. Psychological Review, 117(4), 1144–1167. 10.1037/a0020511, 21038975

[bib89] Seehagen, S., & Herbert, J. S. (2011). Infant imitation from televised peer and adult models. Infancy, 16(2), 113–136. 10.1111/j.1532-7078.2010.00045.x, 32693524

[bib90] Simon, H. A. (1956). Rational choice and the structure of the environment. Psychological Review, 63(2), 129–138. 10.1037/h0042769, 13310708

[bib91] Shutts, K., Banaji, M. R., & Spelke, E. S. (2010). Social categories guide young children’s preferences for novel objects. Developmental Science, 13(4), 599–610. 10.1111/j.1467-7687.2009.00913.x, 20590724 PMC2898520

[bib92] Shutts, K., Roben, C. K. P., & Spelke, E. S. (2013). Children’s use of social categories in thinking about people and social relationships. Journal of Cognition and Development, 14, 35–62. 10.1080/15248372.2011.638686, 23646000 PMC3640585

[bib93] Sutton, R. S., & Barto, A. G. (2018). Reinforcement learning: An introduction (2nd ed.). MIT Press. https://incompleteideas.net/book/the-book-2nd.html

[bib94] Tajfel, H., Billig, M. G., Bundy, R. P., & Flament, C. (1971). Social categorization and intergroup behaviour. European Journal of Social Psychology, 1, 149–178. 10.1002/ejsp.2420010202

[bib95] Tarantola, T., Kumaran, D., Dayan, P., & De Martino, B. (2017). Prior preferences beneficially influence social and non-social learning. Nature Communications, 8(1), 817. 10.1038/s41467-017-00826-8, 29018195 PMC5635122

[bib96] Taylor, M. G. (2013). Gender influences on children’s selective trust of adult testimony. Journal of Experimental Child Psychology, 115(4), 672–690. 10.1016/j.jecp.2013.04.003, 23708732

[bib97] Teh, Y. W. (2010). Dirichlet process. https://www.gatsby.ucl.ac.uk/∼ywteh/research/npbayes/dp.pdf

[bib98] Ullman, T. D., Baker, C. L., Macindoe, O., Evans, O., Goodman, N. D., & Tenenbaum, J. B. (2009). Help or hinder: Bayesian models of social goal inference. In Y. Bengio, D. Schuurmans, J. D. Lefferty, C. K. I. Williams, & A. Culotta (Eds.), Advances in neural information processing systems 22 (pp. 1874–1882). Curran Associates Inc.

[bib99] Vélez, N., Wu, C. M., & Cushman, F. A. (2022). Representational exchange in social learning: Blurring the lines between the ritual and instrumental. Behavioral and Brain Sciences, 45, e271. 10.1017/S0140525X22001339, 36353874

[bib100] Wilkinson, G. S. (1992). Information transfer at evening bat colonies. Animal Behaviour, 44, 501–518. 10.1016/0003-3472(92)90059-I

[bib101] Witt, A., Toyokawa, W., Lala, K. N., Gaissmaier, W., & Wu, C. M. (2024). Humans flexibly integrate social information despite interindividual differences in reward. Proceedings of the National Academy of Sciences, 121(39), e2404928121. 10.1073/pnas.2404928121, 39302964 PMC11441569

[bib102] Wood, L. A., Kendal, R. L., & Flynn, E. G. (2012). Context-dependent model-based biases in cultural transmission: Children’s imitation is affected by model age over model knowledge state. Evolution and Human Behavior, 33, 387–394. 10.1016/j.evolhumbehav.2011.11.010

[bib103] Zhao, B., Lucas, C. G., & Bramley, N. R. (2022). How do people generalize causal relations over objects? A non-parametric Bayesian account. Computational Brain & Behavior, 5, 22–44. 10.1007/s42113-021-00124-z, 34870096 PMC8631267

[bib104] Zhi-Xuan, T., Mann, J. L., Silver, T., Tenenbaum, J. B., & Mansinghka, V. K. (2020). Online Bayesian goal inference for boundedly-rational planning agents. arXiv. 10.48550/arXiv.2006.07532

[bib105] Ziebart, B. D., Maas, A. L., Bagnell, J. A., & Dey, A. K. (2008). Maximum entropy inverse reinforcement learning. In Proceedings of the 23rd National Conference on Artificial Intelligence (Vol. 3, pp. 1433–1438). AAAI Press.

[bib106] Zmyj, N., Buttelmann, D., Carpenter, M., & Daum, M. M. (2010). The reliability of a model influences 14-month-olds’ imitation. Journal of Experimental Child Psychology, 106(4), 208–220. 10.1016/j.jecp.2010.03.002, 20427052

